# Regularities and Anomalies in Neon Matrix Shifts of Hydrogen-Bonded O–H Stretching Fundamentals

**DOI:** 10.1021/acs.jpca.4c03468

**Published:** 2024-08-19

**Authors:** Margarethe Bödecker, Dmytro Mihrin, Martin A. Suhm, René Wugt Larsen

**Affiliations:** † Institute of Physical Chemistry, University of Göttingen, Tammannstrasse 6, 37077 Göttingen, Germany; ‡ Department of Chemistry, Technical University of Denmark, Kemitorvet 206, 2800 Kgs. Lyngby, Denmark

## Abstract

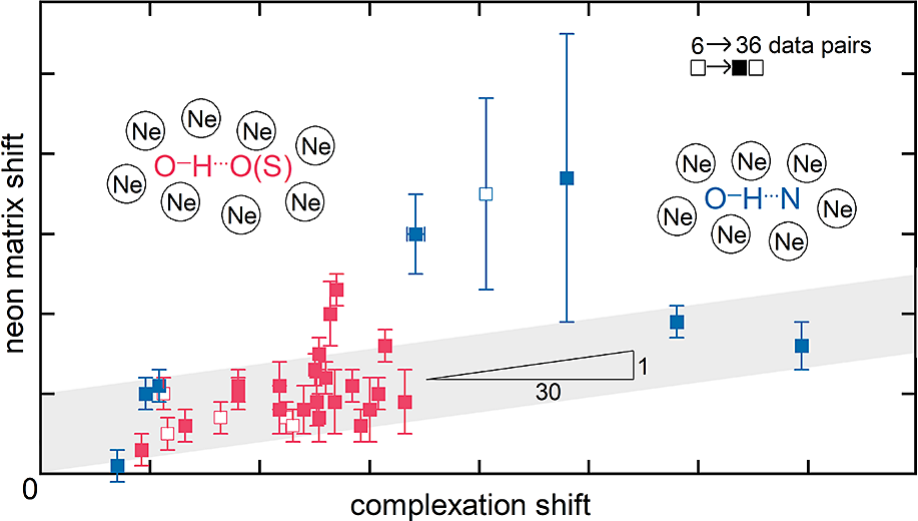

O–H bond stretching vibrations in hydrogen-bonded complexes embedded into cryogenic neon matrices are subtly downshifted from cold gas phase reference wavenumbers. To the extent that this shift is systematic, it enables neon matrices as more universally applicable spectroscopic benchmark environments for quantum chemical predictions. Outliers are indicative of either an assignment problem in one of the two cryogenic experiments or they reveal interesting dynamics or structural effects on the complexes as a function of the environment. We compile 6 literature-known pairs of experimental data in jet and neon matrix expansions and realize a 6-fold expansion of that number through targeted matrix isolation and/or slit jet expansion spectroscopy presented in this work. In many cases, the neon matrix shift is less than the uncertainty of the currently best-performing blind quantum chemical predictions for the gas phase, but in specific cases, it may exceed the currently achievable theoretical accuracy. Some evidence for a positive correlation of the matrix shift with the hydrogen bond shift is found, similar to observations for helium nanodroplets. Outliers in particular for water acting as a donor are discussed, and in a few cases they call for a future reinvestigation. Substantial improvement in the correlation of the matrix shift with the hydrogen bond shift is achieved for ketone monohydrates by removing a vibrational resonance. New insights into nitrile hydration isomerism are obtained, and the linear OH stretching spectrum of the jet-cooled ammonia–water complex is presented for the first time. Vibrational spectroscopy in weakly perturbing solid rare gas quantum matrices for the benchmarking of gas phase theory and future explicit theoretical treatments of the quantum matrix environment to better understand the outliers are both encouraged.

## 1 Introduction

For the empirical performance test of state-of-the-art electronic structure and quantum nuclear dynamics predictions about hydrogen-bonded complexes,^1^ experimental gas phase studies in cold supersonic jets^2^ and traps^3^ are the gold standard. However, they can suffer from restrictions such as sensitivity, indirect spectral probing, or metastable conformations.^4^ Control or even replacement experiments that embed the complex in an inert matrix are highly desirable because they can reduce some of those restrictions by longer observation times, by strictly linear absorption features, and by annealing experiments.^5,6^ The associated spectral matrix interaction shift is not easy to model^7−14^ but often minimized in quantum matrices built from light species such as H_2_, He, or Ne. In contrast, Ar matrices are known for inducing substantial conformational switches, such as in the case of ethanol dimers.^15,16^ Although there are more than a dozen research groups in the field of neon matrix isolation spectroscopy of hydrogen bonding and even more working on corresponding supersonic jet characterizations, there is a surprisingly low number of published OH stretching data pairs which would allow us to judge the influence of the matrix. Here, we extend previous coordinated jet plus Ne-matrix experiments^17−20^ and increase the number of available data pairs from 6 to 36 in three ways: by experimentally complementing single literature values with their missing matrix or cold gas phase counterpart (5 cases); by making available some previously unpublished reference spectra obtained in our groups (22 cases); and by targeting a few new systems with both experimental techniques (3 cases). The size of the expected Ne matrix downshifts is on the order of 10 cm^–1^ for the particularly environment-sensitive O–H stretches, perhaps slightly increasing with increasing hydrogen bond strength. This is comparable to rare residual site splittings in this spectral range, still well below 1% of the absolute transition wavenumber, and it is roughly within explicit anharmonic contributions to O–H stretching fundamentals which cannot be accounted for by simple computational harmonic scaling approaches.^2,21^

New jet/Ne-matrix data pairs and critical analysis of the published literature can lead to a win-win situation: In regular cases, the data pairs allow to identify any systematic trends in such Ne-matrix shifts and thus widen the benchmark potential of spectroscopic experiments toward quantum theory in the field of hydrogen bonding with its mix of intermolecular interactions. In anomalous cases, they may hint at potential spectral interpretation issues on the gas phase or matrix isolation side.^22,23^ Otherwise, they reveal interesting matrix embedding effects on the complex structure and vibrational dynamics, such as conformational switches, tuned anharmonic resonances,^24^ possibly also constrained large amplitude motion or weak cooperative hydrogen bonding to matrix atoms. Indeed, studies of complexes with more classical matrix species M such as N_2_ have previously revealed sizable XH spectral shifts,^25,26^ whenever an XH···XH···M motif is possible. The popular cryogenic tagging technique, which brings action spectroscopy of hydrogen-bonded complexes closer to direct absorption measurements, profits from sufficiently small shifts induced by the tag, which is often chosen to be a typical (quantum) matrix gas.^27^ In rotational spectroscopy, neon complexes are regularly observed and characterized as side products, but definitely worth a closer investigation, whenever they show interesting structures or dynamics.^28^ More often, complexes with heavier rare gas atoms are observed and discussed, and systematic trends across group 18 of the periodic table can be particularly informative.^29^ Therefore, a quantitative understanding of spectral shifts induced by single, multiple, or infinite numbers of rare gas atoms attached to a molecular system has multiple benefits.

After a brief description of the employed experimental techniques and uncertainty assessments, we present an initial table of dominant peak wavenumbers in the gas and Ne matrix phases, categorized according to the spectral technique and resulting in formal band position shifts due to the matrix environment. In the next step, several of these shifts are critically assessed with possible resonance partners or other spectral complications in mind, thus leading to a cautious adjustment and uncertainty estimate of the underlying O–H oscillator position and its shift in the neon matrix. We discuss selected outliers as a function of hydrogen bond strength and type, suggest a reinvestigation in some cases, and end with a bold toy model proposal to trigger theoretical and experimental extensions of this work.

Given that O–H stretches are particularly sensitive to tagging^30^ and matrix effects,^26^ the present study probably comes close to the worst-case scenario for the use of vibrational matrix data in comparison with gas phase computations. Only acidic X–H stretches are expected to be more sensitive to the matrix environment.^31−33^ We thus conclude that Ne matrix isolation spectroscopy has a substantial benchmark potential^34^ for quantum chemistry and dynamics beyond the hydride stretching region, in particular for the class of large-amplitude intermolecular modes generated by hydrogen bonding, which are observed in the experimentally challenging far-infrared spectral region.^35−37^

## 2 Experimental Section

Direct infrared absorption by molecular complexes in the cold gas phase is a powerful technique to detect the position of hydride stretching fundamentals, often in combination with long slit jet expansions.^38,39^ Here, we employ the most recent variant of an FTIR-probing approach^40^ which uses gas recycling to make expensive compounds and carrier gases accessible despite a high gas throughput. Relevant parameters for the present work include a spectral resolution of 2 cm^–1^ (in explicitly denoted cases 1 cm^–1^ or less), the use of an InSb detector together with an optical filter to address OH stretching fundamentals, the application of a gas stagnation pressure of less than 1 bar, and the use of typical dilutions of hydrogen bond acceptor and donor molecules in a lower than 1:100 ratio to the carrier gas (for specific values see the specific spectral figures, also in the Supporting Information). The pulsed admission of such gas mixtures to a vacuum container through the Bruker VERTEX 70v FTIR-probed 700 mm slit nozzle far exceeds the available pumping capacity of 2000 m^3^/h. This is compensated for by a 4 m^3^ vacuum buffer and a sufficiently long waiting time between pulses. Such a brute-force gas-phase approach is still orders of magnitude less sensitive than matrix isolation, which is again combined with FTIR spectroscopy in the present work. This experimental setup employs a 4K liquid helium-free closed-cycle cryocooler from Advanced Research Systems interfaced with a Bruker VERTEX 80v FTIR spectrometer.^37^ In brief, doped matrices of neon are prepared by mass flow-controlled deposition of LN2 precooled neon gas onto a wedged diamond window housed in an oxygen-free high-conductivity copper window holder attached to the cold head. The outer rotatable vacuum shroud of the cryocooler is equipped with two additional wedged diamond windows, providing an optical port for combined infrared and terahertz investigations. An independent dual inlet copper tubing system enables both the codeposition of selectively isotopically enriched samples and spatial “spot-to-spot” investigations of solute-rich and water-rich regions of the doped neon matrices.^41,42^ A combination of resistive heaters, Si diode temperature sensors, and feedback electronics furthermore promotes the option to anneal the doped neon matrices up to 9.5 K. This annealing procedure both circumvents rare site effects and triggers further complexation events in the soft neon environment. A spectral resolution of 0.6 cm^–1^ is often chosen as the best compromise between the signal-to-noise ratio and sufficient spectral resolution to resolve observed band structures. For the neon matrix isolation spectra, the mixing ratios are less important than the spatial distribution of the dopants within the matrix, which is monitored by the 3.5 mm IR probe beam. This is due to the separate deposition inlet tubing approach, which has been designed for partial D-enrichment investigations. The distributions of dimeric and trimeric species differ significantly spatially within the approximately 10 × 10 mm^2^ circular matrices even for a specific mixing ratio. This allows for the easy distinction between monomeric, dimeric, and trimeric species within the same experiment. Furthermore, a series of spectra have been collected after annealing to 9.5 K along with independent complementary reference spectra for each of the subunits. The combined data sets are often sufficient to confidently establish the dimer assignments. In both experiments, decadic absorbance spectra are collected by reference to a background spectrum without gas flow or deposited matrix. These absorbances are in the range of 1 for the neon matrix spectra but only 10^–4^–10^–5^ in the jet spectra, thus underscoring the desire to replace jet studies with matrix isolation studies for systematic benchmarking work. Used chemicals are listed in Table S4.

Normally, it is sufficient to determine the band maximum of a vibrational transition in either the jet or the matrix spectrum for a neon matrix shift calculation, given the modest asymmetry of the observed bands with a typical width (FWHM) between 2 and 10 cm^–1^ and the integer wavenumber precision targeted in this work. One would then assign a base uncertainty of ±1 cm^–1^ to the observed shifts, which may already arise from limiting integer rounding effects. The spectral intensity, which is often harder to quantify, would not be relevant in the shift determination. However, in several cases involving water complexes, there are anharmonic resonances^43−45^ which may be tuned or detuned by the matrix environment. The nature of these resonances is that a dark state with essentially no intrinsic IR intensity (typically less than 5% of the total intensity involved) steals the intensity from the bright OH stretching state by wave function mixing. In this dark state model limit, the observed intensity is spread over two or more signals, but the position of the original OH bright state can still be obtained in a good approximation by working out the center of the intensity spread over the resonance partners. This conservation of the center of spectral intensity follows from the trace invariance upon diagonalization of an effective normal mode coupling matrix. It may not hold for vibrational Franck–Condon progressions.^46,47^ Nevertheless, such an effective low-dimensional Hamiltonian model may be the best one can do in the absence of a rigorous anharmonic treatment, as it would be possible for water-dimer-sized complexes.^48,49^ Beyond simply locating the OH stretching state with the strongest transition observed in the jet or in the matrix (which can jump discontinuously when the interacting states switch their spectral sequence), we thus investigate the shift of the intensity centers of any resonance multiplets wherever these can be clearly identified. For this, we need both the positions and relative intensities of the observed multiplet partners. Our direct absorption spectroscopy techniques are favorable for such an approach. However, there is still considerable uncertainty in the analysis of a real spectral trace due to noise, baseline effects, spectral wings, spectral overlap, monomer and cluster impurities, matrix site splittings, annealing effects, or hot band contributions in the jet due to insufficient vibrational cooling. To minimize the arbitrariness of the proposed resonance multiplet analysis, we used a set of pragmatic rules:

1. Ne matrix and jet spectra for a given system are treated on an equal footing when choosing the width of integration windows
2. Classes of compounds such as ketones are treated on an equal footing
3. Uncertainties from noise, baseline effects, and integration window size are propagated from two limiting bandwidth choices

Details on the signal intensity analysis procedure are described in the Supporting Information (Figure S1, Tables S1 and S2).

In situations of limited signal-to-noise ratio (typical for jet spectra), where resonances may be expected but are not detected, we include the potential effect on the uncertainty of a one-sided (and in this sense worst-case) resonance barely hidden in the noise. Specifically, we assume that this hidden resonance is separated from the main signal by twice the typical resonance coupling element *W* (*W* ≈ 10 cm^–1^ for b2lib resonances,^44^*W* ≈ 30 cm^–1^ for b2ON resonances^45^ and *W* ≈ 50 cm^–1^ for OH bend overtone (b2) resonances^45,50^). The threshold wavenumbers below which such water-specific resonances may become relevant are chosen as 3550 cm^–1^ (b2lib), 3450 cm^–1^ (b2ON) and 3350 cm^–1^ (b2), respectively. This increases the uncertainty of the intensity center for water complexes compared to the uncertainty of a single absorption band, even in the absence of any other spectral feature. For details, see Table S3 in the Supporting Information. In matrix spectra, the signal-to-noise ratio is typically less limiting than residual site splittings or impurity bands from other aggregates. For literature Ne matrix values of water complexes, we typically assume a rough uncertainty of 10 cm^–1^ for the intensity center. For Ne matrix spectra obtained in this work, more specific analyses can be carried out based on the stochastic integration procedure.^51^

An error source that is difficult to quantify involves possible CH combination bands of the acceptor molecule underneath the signals assigned to the OH stretching fundamental and its resonance partners. These could distort the relative intensities and thus the intensity center obtained by deperturbation, in particular, for large residues and high acceptor concentrations. Variation of the donor/acceptor concentration ratio (if possible down to pure acceptor spectra) can help to rule out major distortions from such non–OH signals in the jet and matrix spectra.

## 3 Results

We start with a compilation of hydrogen-bonded OH stretching fundamentals for the available pairs of (He or Ne) jet-cooled and (Ne) matrix-cooled hydrogen-bonded 1:1 complexes, including both literature data and results reported in this work (TW) for the first time. Where the intensity of the OH stretch is believed to be redistributed among several bands due to resonances, we list only the dominant transition (raw). Table 1 summarizes hydrogen bonds to chalcogen atoms (OH–O(S)) and Table 2 lists OH–N contacts.

**Table 1:** Raw Wavenumber Downshifts Δ of Dominant O–H Stretching Vibrations in the Ne Matrix Compared to the Gas Phase Values for Different OH–O(S) Donor–acceptor Pairs Using IR-BD (Beam Depletion), CRD (Cavity Ring-down), fs-IR (Femtosecond Ionization Coupled with IR), IR-UV (Double Resonance, Including IR-REMPI Variants), and FTIR Techniques^a^

OH–O(S) species	jet experiment	ν̃_jet_	ν̃_mat_	matrix experiment	Δ
w–2,2,2-trifluoroacetophenone	FTIR slit^2^	3611(1)	3608(1)	Ne matrix^TW^	3(2)
w–w	IR-BD^52^	3601(1)	3591(1)	Ne matrix^53^	10(2)
	CRD^54^	3600			
	Raman^55^	3602			
	fs-IR^56^	3601			
	FTIR slit^44^	3602			
w–diacetyl	FTIR slit^20^	3599(1)	3594(1)	Ne matrix^20^	5(2)
w–diacetyl (*)		3575(1)	3568(1)		7(2)
w–formaldehyde	FTIR slit^2^	3591(1)	3585(1)	Ne matrix^TW^	6(2)
methanol–methanol	CRD^57^	3574(1)	3567(1)	Ne matrix^58^	7(2)
	FTIR slit^59^	3575			
w–methanol	FTIR slit^60^	3567(1)	3556(1)	Ne matrix^TW^	11(2)
w–cyclobutanone	FTIR slit^44^	3548(1)	3540(1)	Ne matrix^TW^	8(2)
w–ethanol	FTIR slit^60^	3548(1)	3537(1)	Ne matrix^TW^	11(2)
w–oxirane	FTIR slit^18^	3542(1)	3536(1)	Ne matrix^18^	6(2)
w–acetone	FTIR slit^44^	3538(1)	3515(1)	Ne matrix^TW^	23(2)
w–2-propanol	FTIR slit^TW^	3537(1)	3529(1)	Ne matrix^TW^	8(2)
w–acetophenone	FTIR slit^44^	3536(1)	3513(1)	Ne matrix^TW^	23(2)
w–acetophenone (*)	FTIR slit^44^	3567(1)	3557(1)	Ne matrix^TW^	10(2)
w–cyclohexanone	FTIR slit^44^	3532(1)	3510(1)	Ne matrix^TW^	22(2)
ethanol (g)–ethanol (g)(^★^)	FTIR slit^16^	3531(1)	3519(1)	Ne matrix^TW^	12(2)
	Raman^61^	3532			
w–*t*-butanol	FTIR slit^62^	3530(1)	3523(1)	Ne matrix^TW^	7(2)
phenol–phenol	IR-UV^63^	3530(1)	3515(1)	Ne matrix^TW^	15(2)
			3507	Ne matrix^64^	
methanol–*t*-butanol	FTIR slit^65^	3529(1)	3513(1)	Ne matrix^TW^	16(2)
phenol–w	IR-UV^66,67^	3522(1)	3499(1)	Ne matrix^TW^	23(2)
	Raman^68^	3523			
	IR-UV^69,70^	3524			
w–cycloheptanone (^★^)	FTIR slit^44^	3512(1)	3504(1)	Ne matrix^TW^	8(2)
w–tetrahydrothiophene	FTIR slit^2^	3507(1)	3499(1)	Ne matrix^TW^	8(2)
w–cyclooctanone (^★^)	FTIR slit^2^	3503(1)	3493(1)	Ne matrix^TW^	10(2)
w–cyclooctanone (*)(^★^)	FTIR slit^2^	3525(1)	3505(1)	Ne matrix^TW^	20(2)
*t*-butanol–*t*-butanol	FTIR slit^65^	3497(1)	3491(1)	Ne matrix^TW^	6(2)
w–tetrahydrofuran	FTIR slit^2^	3491(1)	3482(1)	Ne matrix^TW^	9(2)

a All numbers are in cm^–1^. TW = This work. w = H_2_O. Largely sorted in the sequence of decreasing wavenumber without separating metastable isomers (*). Systems, where a conformational switch upon matrix embedding could potentially be happening, are marked with (^★^). If TW data are not discussed in the main document, the experimental data can be found in Figures S4 and S6–S17 in the Supporting Information.

**Table 2:** Raw Wavenumber Downshifts Δ of Dominant O–H Stretching Vibrations in the Ne Matrix Compared to the Jet-Isolated Values for Different OH–N Donor–acceptor Pairs Using IR-UV (Double Resonance, Including IR-REMPI Variants) and FTIR Techniques^a^

OH–N species	jet experiment	ν̃_jet_	ν̃_mat_	matrix experiment	Δ
w–acrylonitrile	FTIR slit^TW^	3622(1)	3621(1)	Ne matrix^71^	1(2)
w–acrylonitrile (*)	FTIR slit^TW^	3609(1)	3599(1)	Ne matrix^71^	10(2)
w–acetonitrile	FTIR slit^TW^	3603(1)	3592(1)	Ne matrix^53^	11(2)
2-aminoethanol (M)	FTIR slit^72^	3568(1)	3554(1)	Ne matrix^TW^	14(2)
	FTIR slit^73^	3569			
w–ammonia	FTIR slit^TW^	3486(1)	3456(1)	Ne matrix^74^	30(2)
	IR-UV^75^	≈3485			
	gas phase^76^	3515			
w–pyridine	FTIR slit^2^	3454(1)	3419(1)	Ne matrix^77^	35(2)
	gas phase^76^	3480			
w–methylamine	FTIR slit^TW^	3417(1)	3380(1)	Ne matrix^43^	37(2)
methanol–methylamine	FTIR slit^TW^	3396(1)	3377(1)	Ne matrix^TW^	19(2)
methanol–dimethylamine	FTIR slit^TW^	3339(1)	3323(2)	Ne matrix^TW^	16(3)
	IR-VUV^78^	3334			
2-aminoethanol–2-aminoethanol	FTIR slit^72^	3304(1)	3287(1)	Ne matrix^TW^	17(2)
	FTIR slit^73^	≈3300			

a All numbers in cm^–1^. TW = This work. (M) stands for internally hydrogen-bonded monomers. w = H_2_O. Sorted in the sequence of decreasing wavenumber without separating metastable isomers (*). The experimental data on methylamine monohydrate and 2-aminoethanol are available from Figures S3 and S5 in the Supporting Information.

The tables include 8 new jet measurements and 24 new neon matrix data to maximize the number and diversity of available pairs. In the final columns of Tables 1 and 2, the resulting matrix downshift in cm^–1^ is listed together with its estimated error bar. We use integer numbers throughout because of the limited spectral resolution and unresolved band contours in most gas phase data and the dependence on annealing conditions and other heterogeneities in the matrix. We are not aware of any known cases of conformational switching induced by neon matrix embedding. Still, for a few systems, conformational switching upon matrix embedding in the sense of one conformation disappearing and another one appearing upon matrix embedding (both within a monomer and with respect to the contact point between monomers) may be happening (see (^★^) in Table 1). These cases are discussed in more detail in the Supporting Information (Figures S7 and S10–S12). Future positive evidence for such a neon-matrix-induced conformational switch in a hydrogen-bonded complex would provide a welcome testing ground for any models describing regular matrix effects.

Wherever the infrared OH stretching intensity of a water complex is found or even expected to spread to resonance partners (in 16 cases), we apply an appropriate deperturbation strategy or at least deperturbation uncertainty based on assigned or potential resonance partners^44,45^ and report the results in Table 3. The deperturbation relies on a negligible intrinsic intensity of the resonance partners, usually overtone or combination bands, and will be exemplified later. It also relies on the assumption that the redistributed intensity arises from OH stretching wave function mixing into dark states, rather than from Franck–Condon-like progressions which do not involve wave function mixing.

**Table 3:** Deperturbed Wavenumber Downshifts Δ of Water (w) Hydrogen-Bonded O–H Stretching Vibrations in the Ne Matrix Compared to the Cold Gas Phase Values for Different OH–O(S) and OH–N Donor–acceptor Pairs Using FTIR Techniques^a^

	ν̃_jet_	ν̃_mat_	Δ
OH–O(S) species			
w–cyclobutanone	3548(2)^44^	3540(1)^TW^	8(3)
w–ethanol	3548(2)^60^	3537(1)^TW^	11(3)
w–oxirane	3542(1)^18^	3536(1)^18^	6(2)
w–2-propanol	3537(2)^TW^	3529(1)^TW^	8(3)
w–acetone	3532(1)^44^	3519(1)^TW^	13(2)
w–acetophenone	3531(2)^44^	3522(1)^TW^	9(3)
w–*t*-butanol	3530(2)^62^	3523(1)^TW^	7(3)
w–cyclohexanone	3523(3)^44^	3514(1)^TW^	9(4)
w–cycloheptanone	3515(1)^44^	3504(1)^TW^	11(2)
w–tetrahydrothiophene	3507(3)^2^	3499(1)^TW^	8(4)
w–cyclooctanone	3503(1)^2^	3493(1)^TW^	10(2)
w–cyclooctanone (*)	3525(3)^2^	3505(1)^TW^	20(4)
w–tetrahydrofuran	3491(3)^2^	3482(1)^TW^	9(4)
OH–N species			
w–ammonia	3486(4)^TW^	3456(1)^74^	30(5)
w–pyridine	3454(2)^2^	3419(10)^77^	35(12)
w–methylamine	3417(8)^TW^	3380(10)^43^	37(18)

a All numbers in cm^–1^. TW = This work. w = H_2_O. Sorted by decreasing wavenumber but keeping isomers (*) together. A list of all data pairs with water as the donor is given in Table S6 in the Supporting Information.

All of the Ne matrix shifts in Table 1 (OH–O(S)) are downshifts, and most are within 15 cm^–1^, including their uncertainty. The only outliers are phenol when donating a hydrogen bond to water and a few ketone monohydrates with OH-stretching wavenumbers near 3500 cm^–1^, which are explained below.

A rather different situation is found for nitrogen as hydrogen bond acceptor, as shown in Table 2 (OH–N). The most prominent matrix downshifts are caused by the monohydrates of pyridine and amines, including ammonia. In contrast, the studied methanol complexes appear to shift significantly less.

The smallest neon downshift of any hydrogen-bonded system, in fact, a negligible one is found for acrylonitrile monohydrate but only for the isomer in which the water coordinates the nitrogen side-on, exposing the nitrogen lone pair to the matrix. Such small or even negative neon matrix downshifts are quite common for the studied OH donor molecules without a hydrogen bond partner. Available values are summarized in the Supporting Information (Table S5).

**Figure 1:**
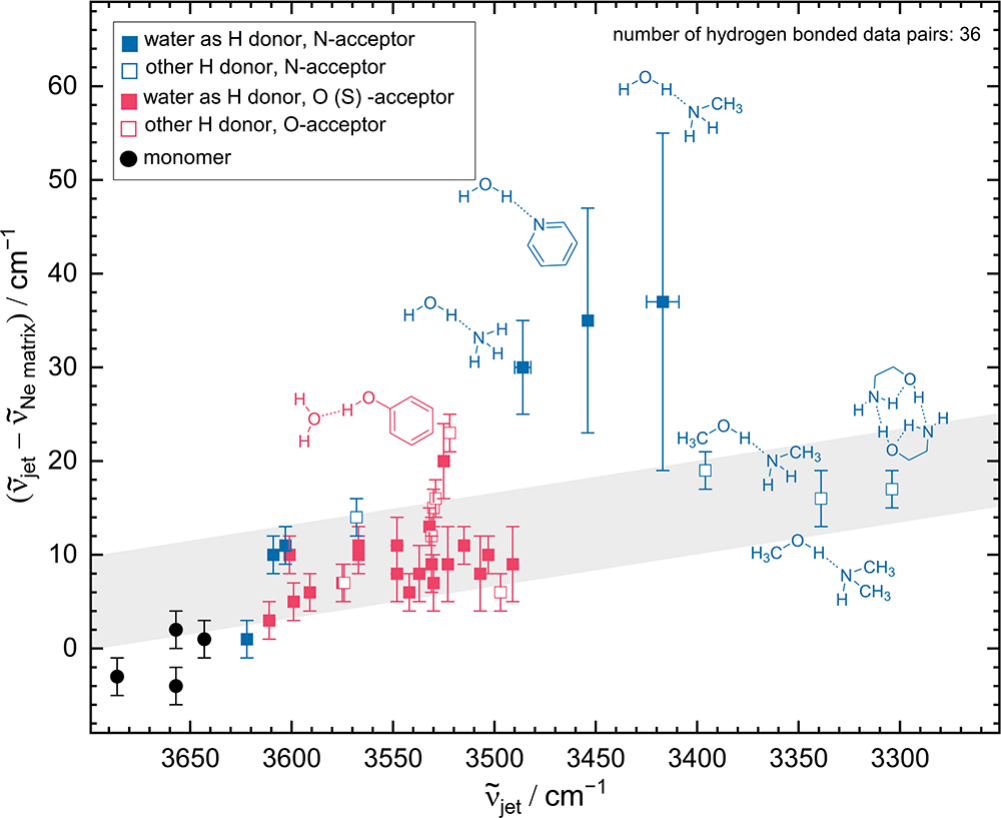
Deperturbed Ne matrix shifts plotted against the hydrogen-bonded OH stretching jet wavenumber for 36 dimers or internally hydrogen bonded monomers (and in black for 4 free monomers that act as donors in the dimers). See Table S6 for the entries.

Figure 1 summarizes the key empirical findings of this work by plotting the neon matrix shifts as a function of the observed jet wavenumber (see Table S6 for the entries). In this figure, deperturbation of the jet or matrix spectra with respect to identified or suspected anharmonic resonances is already taken into account as far as possible. The corresponding figure prior to deperturbation, a deperturbed variant that plots matrix shifts as a function of hydrogen bond-induced downshift rather than absolute jet wavenumber on the abscissa (for intermolecular hydrogen bonds) and one variant only containing literature-known data pairs can be found in the Supporting Information (Figures S18–S20). An alternative would be to plot the matrix shift against the enthalpy of proton transfer.^79^ In black, some relevant non-hydrogen-bonded monomer results are given for comparison and show small shifts of varying sign. The depicted matrix downshifts for hydrogen-bonded systems (red from Table 1 and blue from Table 2) are uniformly positive and most data points scatter within a 10 cm^–1^ wide guiding band with a small positive slope of 1/30. This guiding band basically assumes that a hydrogen bond downshift of 30 cm^–1^ induces an extra matrix downshift of roughly 1 cm^–1^. Such an empirical, approximately linear relationship has been discussed before for hydrogen-bonded complexes in helium nanodroplets.^80^ For neon matrices, the relationship needs to be further explored and refined in the future by adding more examples. However, as mentioned when the corresponding data tables are introduced, there are some pronounced outliers from it, mostly involving water. Some of these outliers will be discussed in the following, and several of them are resolved by the deperturbation strategy.

### 3.1 Discussion of Outliers

#### 3.1.1 Phenol Monohydration

**Figure 2:**
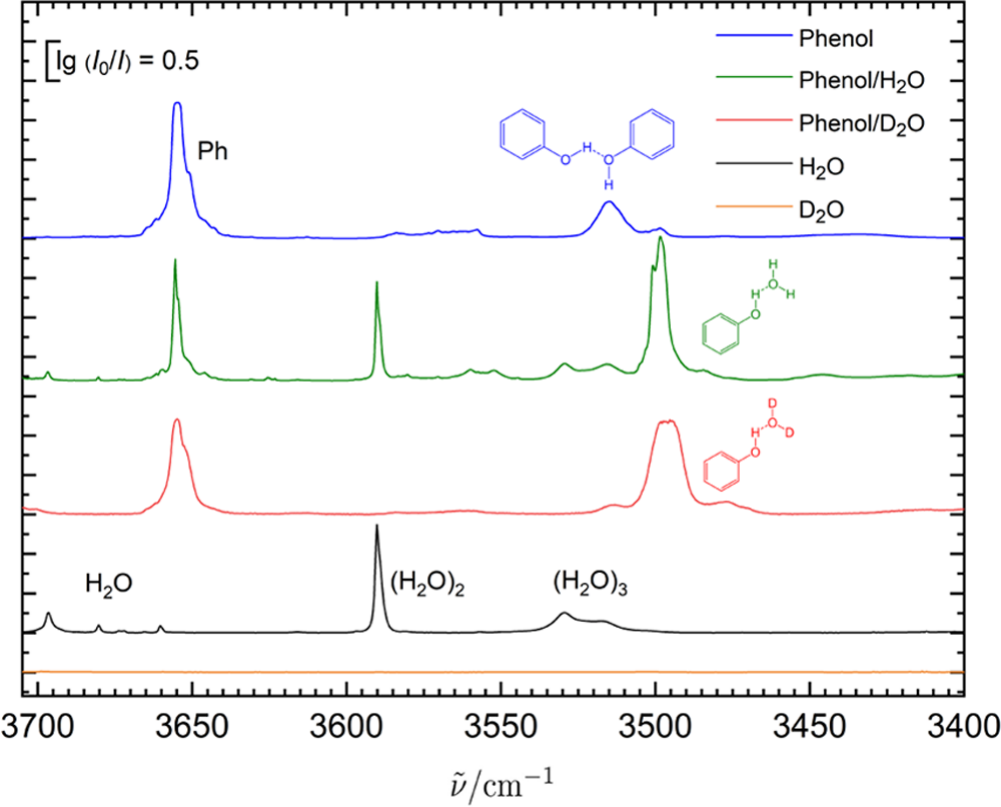
Series of Ne-matrix experiments identifying the phenol OH stretching vibration in its monohydrate among homocluster signals and proving that water is the hydrogen bond acceptor by isotope substitution.

One of the largest matrix shifts in the table of OH–O(S) (Table 1) bonded systems (red symbols in Figure 1) involves the monohydrate of phenol. In the gas phase, it has been studied several times^66−70^ and it is consensus that phenol preferentially acts as a hydrogen bond donor toward water. To ensure that this is also the case in the Ne matrix, Figure 2 summarizes a series of matrix experiments, including individual deuteration of the water component only, which would be difficult to realize in a jet experiment. A more generally applicable but expensive alternative is to use oxygen isotopes, which is feasible for both jet^65^ and cryogenic matrix isolation experiments.^81^ The isotope-edited matrix isolation experiments for phenol monohydrate show that the assigned OHb signal is indeed due to a phenol vibration. Because this vibration depends in a subtle way on the deuteration level of the accepting water (0, 1, or 2 D atoms), the band shifts slightly and broadens somewhat, but deuterated water as a donor would absorb in the entirely different OD stretching region. The figure shows the absorptions of both hetero- and homodimers and traces of larger clusters together with ro-vibrational transitions of water molecules.^82^ Compared to the jet spectra, the matrix bands are only somewhat broader and, at best, slightly split. The peak positions and the band integrals are sufficiently well-defined for the purpose of this work, and the signal-to-noise ratio is much better than in typical jet experiments (vide infra). Therefore, any uncertainty in the matrix spectra of the heterodimers is likely to arise from possible spectral overlap with homodimers and larger clusters.

It remains to be shown by appropriate theoretical calculations why the Ne matrix shift of the phenol monohydrate is more than twice as large as that of cycloheptanone monohydrate or *t*-butyl alcohol monohydrate, where water acts as a donor and the OHb fundamental vibration is similar in wavenumber. However, the intermediate value for phenol dimer (see Table 1, we use our own matrix value rather than the one reported elsewhere^64^ which deviates systematically, also for monomeric phenol) indicates that phenolic OH groups are more susceptible to neon perturbation than water OH groups. This might be related to their planar nature and needs to be explored more systematically.

#### 3.1.2 Ketone Hydrates

**Figure 3:**
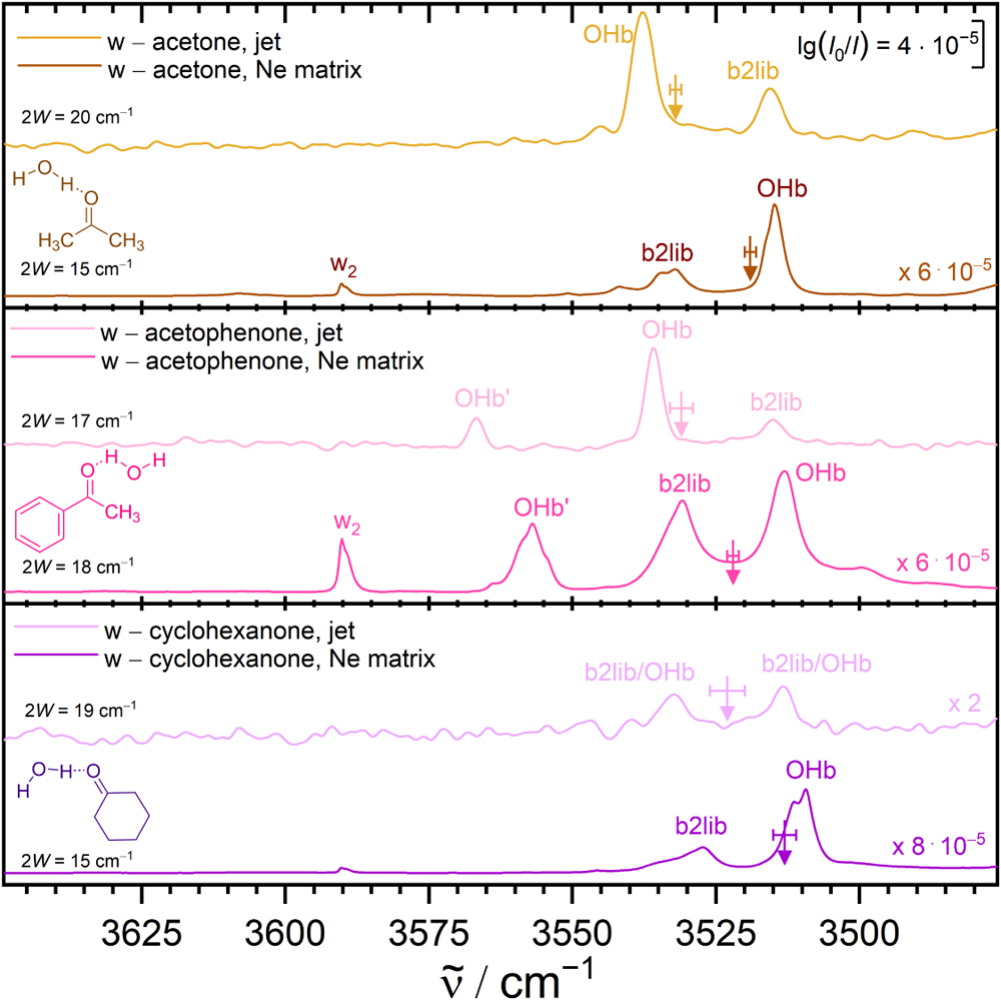
Three cases where the OHb-b2lib resonance is swapped (acetophenone, acetone) or detuned (cyclohexanone) by Ne matrix embedding and a simple resonance treatment regularizes the matrix shift of the OHb mode. The arrows represent the deperturbed band position. *W* = coupling matrix element required to explain the intensity ratio, listed on the left side of the spectra is 2*W*, which corresponds to the distance between the two interacting states when perfectly in resonance (e.g., in the second-lowest trace). See text for further explanations.

The recently discovered^44^ systematic resonance between the water OHb fundamental and a three-quantum state involving the water bending overtone (b2) and a librational fundamental (lib) in ketone monohydrates with an effective coupling matrix element *W* of about 10 cm^–1^ offers an ideal playground for matrix isolation effects. Depending on the substitution pattern of the ketone, the OHb fundamental will be more or less downshifted, whereas the three-quantum state remains remarkably stationary slightly above 3500 cm^–1^ in the investigated jet spectra.^44^ For ketones with an OHb fundamental close to that position, subtle matrix shifts can modify or even invert the resonance situation, formally swapping the order of the zero-order states before engaging in the resonance. Figure 3 gives three examples. In the case of acetophenone, there are actually two hydrogen bond isomers depending on whether water docks on the methyl or on the phenyl side. Phenyl docking is somewhat less stable and also features a less downshifted OHb signal (OHb′). In the jet, there is no appreciable resonance with the three-quantum b2lib state in this case, and the neon matrix downshift is regular. Methyl docking of the water molecule leads to a stronger OHb downshift, still above the dark three-quantum state in the gas phase. Therefore, only part of the OHb intensity is transferred to the dark state, and the higher frequency transition is more intense. This changes in the Ne matrix, which further downshifts the OHb transition, whereas it slightly blue-shifts the three-quantum state (which is plausible for a mode including bending and librational character). That happens to such an extent that the two zero-order states come much closer, intensifying the resonance. They actually cross, and as a consequence, the dominant OHb character (and infrared intensity) is observed at the lower frequency. If one only looks at the dominant transitions, this would suggest an unusual Ne matrix shift of more than 20 cm^–1^. A simple two-state deperturbation brings the matrix shift of the zero-order OHb positions down below 10 cm^–1^, into a regular range for OH–O(S) bonded systems. To experimentally locate the zero-order OHb positions, it is sufficient to calculate the center or weighted average of the intensity of the participating transitions, assuming that the three-quantum state brings no intrinsic intensity with it. A related case is found for the monohydrate of cyclohexanone (Figure 3, bottom). Here, the OHb-b2lib resonance is almost perfect, with equal intensity in both transitions within the signal-to-noise ratio of the jet experiment. Each of the transitions has about 50% OHb stretching character. Now, the Ne matrix shifts necessarily detune the resonance and they do so in the same direction as in acetophenone. The three-quantum state is somewhat upshifted, the OH stretching vibration downshifted and therefore more intensity accumulates in the lower wavenumber transition. The higher sensitivity in the matrix experiment makes it still possible to capture the relative intensity with some degree of confidence. Without deperturbation, the calculation of the matrix shift is somewhat arbitrary, because the jet reference is unclear. It is either anomalously small or anomalously large (Table 1). With deperturbation, once more, one finds a fairly regular Ne-matrix shift of 10 cm^–1^. The most pronounced swap of the resonance partners with matrix isolation is found for acetone monohydrate (Figure 3, top). The strong-weak pattern of the jet doublet is mirrored to a weak-strong pattern, and the distance between the deperturbed OHb positions (13 cm^–1^) is significantly more regular than the distance between the dominant peaks in the jet and in the matrix (23 cm^–1^).

As a side product and consistency check of the b2lib deperturbation, one may compare the coupling matrix elements *W* required to explain the intensity pattern between the two environments (Figure 3, which lists 2*W* as the closest possible approach to the perturbed states). Interestingly, it appears to decrease somewhat from the jet to the matrix for the aliphatic ketones, whereas the coupling quite visibly increases for the aromatic ketone when moving from the jet to the matrix. This could be a real effect, given the modification of the carbonyl group in conjugation with the π system of the aromatic ring. However, one should also consider an alternative explanation that involves the metastable conformer observed for the aromatic ketone (acetophenone, OHb′). While there is no resonance in the jet, the downshift of OHb′ in the matrix compared to the jet wavenumber may light up an OHb′-b2lib′ resonance, whose resonance partner b2lib′ might then coincide with b2lib in the perturbed spectrum. This would lead to an overestimation of b2lib intensity sharing for the global minimum transition and hence an overestimation of its coupling matrix element. However, to bring down the global minimum acetophenone coupling matrix element to the same size as that of the other ketones in the neon matrix (2*W* ≈ 15 cm^–1^) one would have to invoke a far too large coupling matrix element for OHb′, the metastable conformer signature. Therefore, the most consistent interpretation still involves an unusually strong resonance for OHb in acetophenone monohydrate and no resonance for OHb′, but any partial contribution of b2lib′ intensity to the b2lib signal might slightly increase the deperturbed matrix shift of 9 cm^–1^ for OHb and at the same time also slightly increase the listed 10 cm^–1^ matrix shift of OHb′.

The similar size of regular Ne-matrix shifts and the OHb-b2lib coupling matrix elements (both about 10 cm^–1^) in ketone hydrates thus creates a perfect situation to achieve two things: A deeper clarification of the resonance situation observed in the gas phase and a regularization of the matrix shifts for the purpose of understanding their magnitude. A future theoretical model of ketone monohydrates in neon matrices might be successful even in scaled harmonic approximation if it is compared to deperturbed experimental data. It will be more challenging to model matrix-isolated monohydrates of ketones including higher-order anharmonic effects responsible for the b2lib resonance.

An example where the b2lib resonance presumably also plays a role but cannot be unambiguously disentangled with the available spectroscopic material is the monohydrate of cyclooctanone. It was shown to coexist in two conformations^83^ and was part of the HyDRA blind challenge,^2^ where the focus was on the global minimum conformation, but a metastable conformation was observed in the vibrational jet spectrum as well. In the neon matrix, there is also evidence for both conformations, but now the spectral splitting between the two OH transitions is only 12 instead of 22 cm^–1^ in the jet. Whereas the 10(2) cm^–1^ neon matrix shift of the global minimum structure is regular, the shift of the metastable isomer appears too large. Indeed, there is some intensity in the neon spectrum at higher wavenumber, which might be responsible for the downshift of the main signal of the metastable isomer via a b2lib resonance mechanism. While the situation is too congested for a final clarification, the combination of neon matrix and jet spectra may still serve as a resonance alert in such a case, where the jet spectrum alone^2^ had a regular appearance.

#### 3.1.3 H_2_O–NH_3_

**Figure 4:**
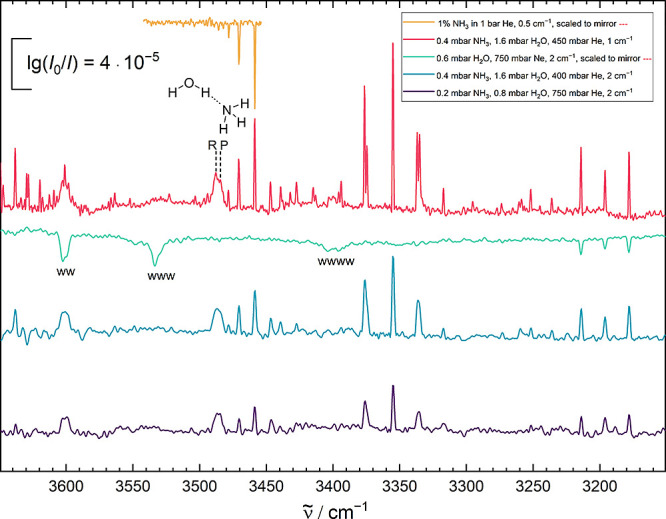
Cold gas phase spectra of ammonia and water (w) with He as the carrier gas at different spectral resolutions and stagnation pressures. The suspected R-branch of ammonia monohydrate peaks at 3488 cm^–1^ and the suspected P-branch at 3484 cm^–1^. By comparing the mixed spectra with an ammonia-only spectrum (yellow, inverted) in the region of the heterodimer signal, the ammonia monomer signals are identified. The positions of water oligomer bands are labeled with w^*n*^ (*n* ≤ 4), in a water-only spectrum (green, inverted, expanded in Ne, and enhanced clustering) to show the low concentration of water trimers and tetramers in the mixed expansions. The three lines around 3200 cm^–1^ are caused by the water monomer bending overtone.

The lightest strongly hydrogen-bonded monohydrate in Table 2 is the complex of water with ammonia. Despite its prototype character which makes it suitable for high-level theory treatments and the expected large amplitude zero-point motion of the 5 hydrogen atoms around the pseudodiatomic O–N frame, there is little known about the OH stretching fundamental. In the context of an important experimental dissociation energy study,^75^ which found preferential energy flow out of the OH stretch excitation into the symmetric (umbrella) NH bend, but not into the OH bend or asymmetric NH bend, a first IR action spectrum was reported. It involved two rotationally unresolved bands, with the higher frequency one remaining somewhat unexplained. Our linear absorption spectra (Figure 4) do not find the higher-frequency band and support the previous assignment of the lower-frequency band to the OH stretching mode. We tentatively resolve a PQR-like structure that is consistent with a ≈10 K rotational temperature and an exponential decay time of >5 ps (corresponding to a Lorentzian FWHM profile of <1 cm^–1^). For significantly shorter lifetimes, the contour would not gain structure when switching from our standard 2 cm^–1^ resolution to 1 cm^–1^. The previously reported band center^75^ (3485 cm^–1^) is nicely confirmed within the joint uncertainties of the two experiments, at 3486 cm^–1^.

The corresponding feature in the Ne matrix spectra^74^ is remarkably downshifted from the gas phase value (Table 2), without any evident nearby resonance partner in the jet and no reported resonance partner in the matrix. The lack of evidence for a resonance is consistent with the experimentally observed energy redistribution,^75^ which does not involve water bending motion. However, it is still conceivable that a resonance partner is hidden underneath the other absorptions in the relatively dense spectra. This could also be true in neon matrix isolation, where only the peak wavenumber is listed.^74^ It may be worthwhile to further investigate this particularly simple species in the neon matrix and to explore isotope substitution. Nevertheless, ammonia serves as a first indicator that NH groups coordinated by OH groups might lead to unusually large neon matrix effects, even for relatively modest hydrogen bond shifts.

#### 3.1.4 Other Amine Hydrates

Adding methyl groups to ammonia has a major impact on the strength of the hydrogen bond. Therefore, it is of interest to see how the unusually large matrix shift of the monohydrate of ammonia (vide supra) evolves with increasing methylation. We leave out trimethylamine because the literature is controversial on both sides (jet spectroscopy^84^ and matrix isolation^85^) and will be the subject of a separate study, as will the monohydrates of dimethylamine and of tertiary amines.^45^

The jet spectrum of methylamine monohydrate has a limited signal-to-noise ratio but is surprisingly simple (Figure S3). Besides slight baseline undulations in the region where one might suspect a weak resonance partner (see Section 2), it exhibits a single strong band at 3417 cm^–1^, indicative of stronger hydrogen bonding than to ammonia. Due to the poor signal-to-noise ratio which may hide a resonance peak, we apply an uncertainty of ±8 cm^–1^ to the center of the OH intensity. ^18^O substitution makes sure the band is due to OH and not NH stretching because it is downshifted by the expected amount. The neon matrix spectrum of methylamine monohydrate has been published before^43^ and shows a significant downshift to 3380 cm^–1^ as well as significant b2 intensity stealing (quantified by the authors at about 20%). Rather than attempting a deperturbation based on published intensities or an experimental reinvestigation targeting the resonances, which we reserve for a later study, we apply a tentative uncertainty of 10 cm^–1^ to the literature neon matrix value. Although the effect of the reported b2 intensity would be to increase the matrix shift of the intensity center, there might be a compensating effect from a b2ON state, like in tertiary amines.^45^ In this way, very much like ammonia, methylamine monohydrate is indicative of an unusually large neon shift of amine hydrates but requires further investigation to narrow down the error bar.

A similar statement can be made for pyridine monohydrate, if one takes the clear-cut single jet transition^2^ at 3454 cm^–1^ and compares it to a neon matrix assignment^77^ of 3419 cm^–1^, with an estimated uncertainty of ±10 cm^–1^ due to the congested nature of the matrix spectrum. Though uncertain, it is the third nitrogen acceptor that appears to produce a relatively large neon matrix shift in the water donor OH position, which is worth being reinvestigated.

#### 3.1.5 Replacing Water by Methanol

**Figure 5:**
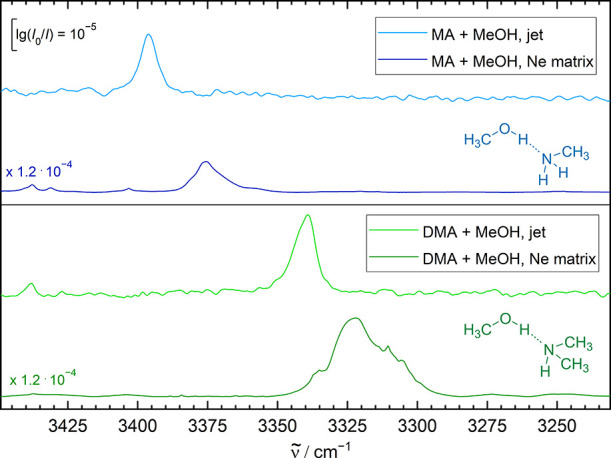
Jet and Ne matrix infrared spectra of methylamine (MA) and dimethylamine (DMA) complexed by a single methanol molecule. The dominance of a single transition demonstrates the absence of anharmonic resonances in the methanol complexes. This facilitates the shift analysis compared to monohydrates of amines, which will be the subject of a future study.

An important clue for the unusually large water neon matrix shifts indicated by literature values in combination with the current jet results comes from the replacement of water by methanol. This removes some resonance opportunities because the OH bending mode and its overtone (b2) now shift to a lower energy. Indeed, the spectra of methanol–methylamine and methanol–dimethylamine (Figure 5) do not show evidence for resonances and their neon matrix shifts are now down to more normal values (Table 2).

This indicates that the anomalous shift found for methylamine monohydrate may be specific for the light water molecule, potentially due to the large amplitude librational motion of the hydrogen bond, which may be more affected by the neon environment than in the more bulky and heavy methanol case. In the future, this calls for a more systematic investigation of different amine monosolvates.

An important role in such a reanalysis could be played by DOH, which binds to the amines almost in the same way as H_2_O does. However, monodeuteration removes the b2 resonance option, so that one expects single bands near the HOH features, which might be easily compared between jet and matrix spectra. If the matrix shift of DOH toward amines is close to that of methanol, the special resonance possibilities of HOH might be responsible for the shift anomaly. If it is close to that of HOH, then the accessibility of neon atoms to strong OH···N hydrogen bonds may be a relevant factor. However, due to the expected metastability^86^ of DOH···N arrangements in comparison to HOD···N coordination (in particular during the long observation times in a matrix) and the accompanying deuteration of nontertiary amines, it is not clear whether monodeuteration will be a viable strategy.

Any deeper analysis of amine monohydrate shifts in neon matrices will need to encompass the more bulky tertiary amines, which have revealed strong resonances in the cold gas phase^45^ but also offer strong downshifts which will help identify a systematic correlation between hydrogen bond shifts and matrix shifts.

#### 3.1.6 Nitrile Hydrates

**Figure 6:**
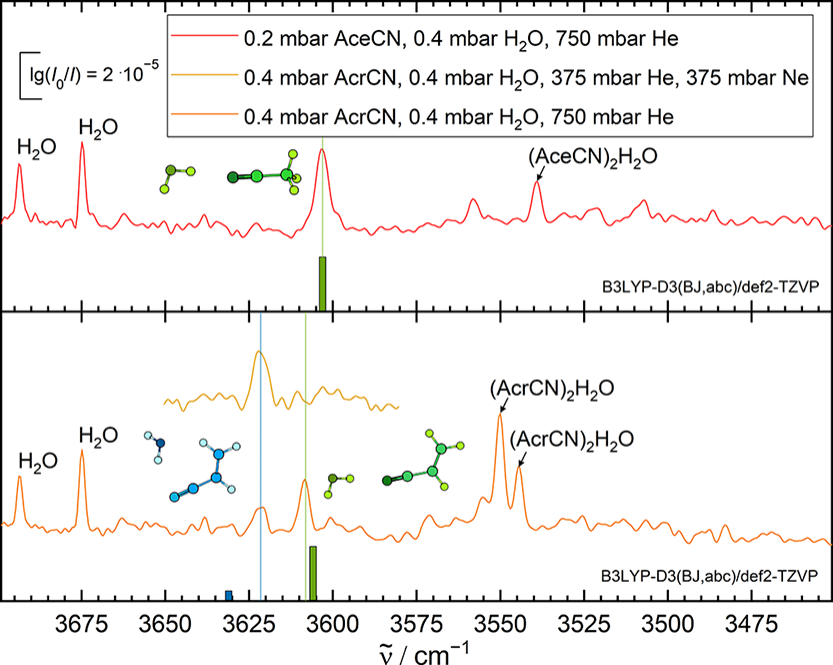
FTIR jet spectra of the OH stretching region of water–nitrile clusters. While for water–acetonitrile (AceCN, top) only one conformer was observed, two conformers are observed for water–acrylonitrile (AcrCN, bottom) in He as a carrier gas. The admixture of Ne results in depopulation of the metastable conformer. The bars below the spectra indicate relative harmonic wavenumber and intensity predictions (B3LYP-D3(BJ,abc)/def2-TZVP level of theory, using Orca,^87^ more details in Table S9), wavenumber-scaled by 0.9702 to match the single AceCN band. A more detailed figure is available from the Supporting Information (Figure S2).

The microhydration of nitriles is more subtle because the water interaction is weaker and there is a range of possible coordination geometries involving either the sp-hybridized lone pair of the nitrogen or the π-cloud of the triple bond. The latter allows for some secondary interaction of the water oxygen with an adjacent C–H bond and thus depends on the residue attached to the cyano group. Spectroscopically, they are easy to distinguish because the linear lone pair coordination gives rise to a much larger intensity enhancement and stronger downshift than the bent π-cloud arrangement.^71^ For acetonitrile, the Ne matrix shift of the observed OH stretching band is in the expected range, and it is plausible to assign the theoretically favored lone pair coordination in both the gas phase and the matrix environment (Figure 6). By spectral analogy, the more downshifted out of two acrylonitrile monohydrate signals can be attributed to the same lone pair coordination. However, now, this coordination type is clearly metastable, as suggested by the addition of neon to the carrier gas and by a microwave structural investigation, which did not observe it.^88^ The persistent higher wavenumber transition corresponds to a stronger overall interaction and has a negligible neon matrix shift. It must correspond to one of the two nonequivalent bent in-plane π-cloud coordinations. In the jet, it is clear that the less strained global minimum structure (shown in Figure 6) is formed. The experimentally observed, anomalously small neon matrix shift for this isomer (Table 2) might be a consequence of exposure of the negatively polarized nitrogen atom to the matrix. Further investigation extending to benzonitrile^89,90^ may clarify the assignment.

#### 3.1.7 Toy Model Capturing O–H···N Matrix Shift Anomalies

It is beyond the scope of this contribution to discuss in detail the possible origins of unusual matrix shifts besides those that can be blamed on tuned anharmonic resonances. Mechanical hydrogen bond compression along the bond direction,^14^ restrictions of the hydrogen bond libration,^80^ mechanical matrix cavity size and shape effects,^7,13^ dielectric effects of the matrix environment,^8^ accessibility for stabilizing neon atoms in close vicinity of the polarized OH bonds, coupling between the IR photons and matrix phonons, matrix preparation protocols and matrix granularity, or specific host–guest interactions such as weak but cooperative hydrogen bonding^26^ are among the conceivable origins. Several of these effects will contribute to the 10 cm^–1^ scatter which is highlighted by the gray ribbon in Figure 1.

**Figure 7:**
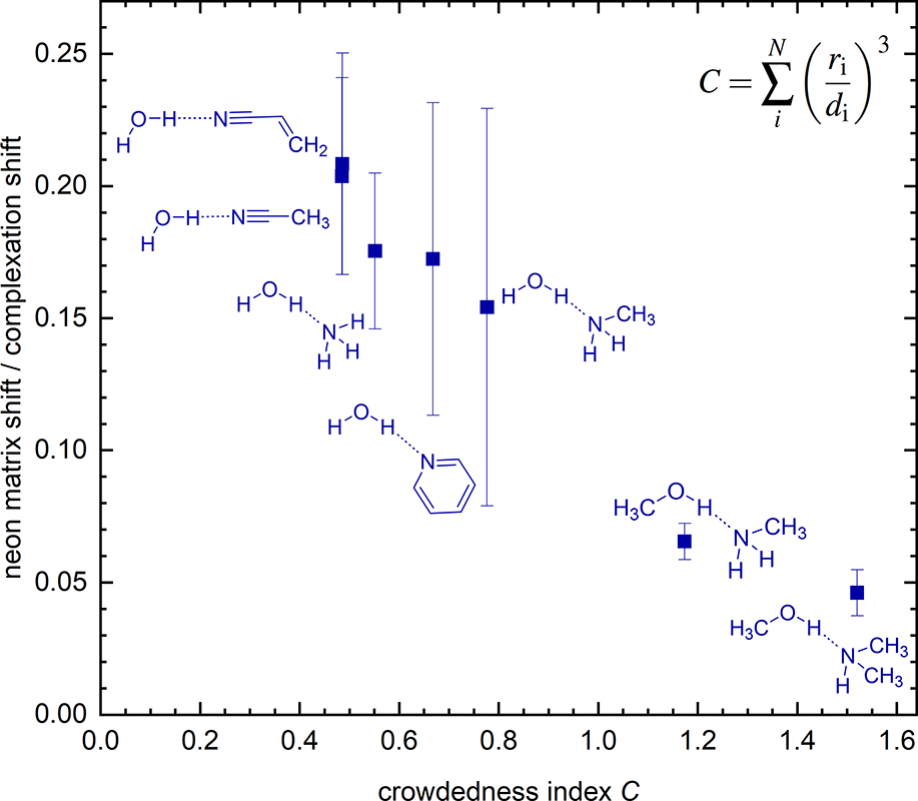
Dimensionless ratio of the observed Ne matrix downshift to the hydrogen bond complexation downshift (donor OH wavenumber – complex OH wavenumber, therefore excluding intramolecular hydrogen bonds) plotted against the computed dimensionless crowdedness index *C* of the complex. More details on *r*_*i*_ and *d*_*i*_ are given in Tables S7 and S8 and all xyz coordinates are shown in Tables S10–S16 in the Supporting Information.

We would just like to introduce a simple toy model that might provide a starting point for the future discussion of severe outliers. It assumes that the atoms and groups attached to the central O–H···N unit (which seems to cause particularly strong anomalies) partially block the matrix atoms from laterally perturbing the hydrogen bond. By using dispersion-corrected hybrid-DFT calculations to obtain a plausible structure of the hydrogen-bonded complex (for computational details, see Table S9), one can work out distances from the groups and atoms to the central H atom (*d*_*i*_) and sum over the ratio between the van der Waals radius^91^ of the group or atom *r*_*i*_ and this distance *d*_*i*_, after raising it to the power of 3. The resulting simple-minded and pairwise additive crowdedness index *C* = ∑ _*i*_^*N*^ (*r*_*i*_/*d*_*i*_)^3^ can be used as a crude proxy for the accessibility of the hydrogen bond to the matrix environment. By plotting the ratio of the observed Ne matrix shift to the hydrogen bond complexation downshift as a function of *C*, one obtains a surprisingly monotonous correlation (see Figure 7), within error bars. The less accessible the hydrogen bond circumference is to the matrix atoms (assuming that they do not lead to a major distortion from the predicted gas phase structure), the less its OH stretching mode appears to become affected by the matrix. This might help to rationalize why methanol is a more regular hydrogen bond donor than water in the neon matrix, among other observations. The task ahead is to fill this correlation or variants thereof with many more experimental data points and to check whether the relationship is more coincidental or more systematic and is worth being refined.

Further experimental investigations should obviously include bulky groups attached to the OH group and the N acceptor because, in the limit of a fully substituent-embedded strong OH–N hydrogen bond, it appears plausible that the neon matrix shift may again vanish due to the inaccessibility of the matrix host to the interaction center. Such new data pairs will thus be particularly valuable in checking any crowdedness index or alternative concepts.

## 4 Conclusions

Neon matrix shifts are frequently considered to be minor in comparison to uncertainties associated with quantum-chemical calculations, at least in neutral systems. For example, a value typically less than 4 cm^–1^ has recently been quoted for molecules.^92^ For diatomics, a systematic study^93^ revealed small shifts for neon, particularly for diatomic hydrides. In this work, we confirm that many hydrogen-bonded systems stay within about 15 cm^–1^ of the OH stretching gas phase value after neon matrix embedding. In several cases, this regular behavior is achieved only by systematic deperturbation from resonance partners, which would otherwise distort the picture because they act differently in the matrix and vacuum environment. We also report a few preliminary Ne matrix downshifts for OH–N hydrogen bonds which exceed the typical range and are even larger than the binding energy of a Ne dimer,^94^ whether or not its zero-point vibrational energy is included. While such large shifts are not unprecedented, they are usually associated with strong acids such as HF, e.g., in combination with reasonably strong bases like ammonia.^31^ The reported Ne matrix shift of about 109 cm^–1^ for HF···NH_3_ actually refers to a thermal gas phase experiment and would likely shrink upon jet cooling. Our tentative evidence for large neon matrix shifts involving water as a very weak acid strongly bound to certain nitrogen-containing acceptors is subject to further investigation. It remains to be seen whether the replacement of water by R–OH always brings back regular matrix shifts, as indicated by a few examples that we have presented.

Highlights of the present extension of the neon shift database include:

1. The first analysis of complexes as elementary as phenol–water (in the matrix, see Figure 2) or water–ammonia (see Figure 4) and water–acetonitrile (see Figure 6, in direct absorption under jet cooling). These can serve as further training systems in future issues of the HyDRA blind challenge.^2^
2. The consistent removal of matrix shift outliers for ketone hydrates by combining matrix and jet studies with a simple resonance deperturbation model based on intensity centers (see Figure 3). This multi-experimental approach to vibrational resonances promises to become a valuable tool beyond the actual matrix shift topic.
3. The regularization of large matrix shifts through the replacement of microsolvating water by methanol and the associated suppression of stretch-bend Fermi resonance opportunities in strongly hydrogen-bonded amine complexes (see Figure 5). It remains to be seen whether this will also be found for more bulky amine hydrates.^45^

An extension of the present OH stretching database to NH,^95^ FH,^96^ ClH,^32,33^ and other hydrides^97^ would be desirable, but for the time being, OH provides the broadest coverage and variation of complexes and our next goal is to systematically extend the OH–N database into the water Fermi resonance regime^45^ while trying to reduce the uncertainties. This will put the proposed simple toy model to a more rigorous test.

The present data coverage already appears large enough to invite more rigorous attempts to model the matrix shifts, e.g., by polarizable embedding models^98^ or DFT cluster and bulk studies.^99,100^ Because one may expect substantial cancellation between repulsive and attractive interaction components in a matrix environment, it appears imperative to have both systematic behavior and outliers included in the growing data set. For this purpose, it would be valuable to build a living database of OH stretching spectra, which are known under both jet cooling and neon matrix isolation conditions. If more such spectral pairs become available in the literature (or if we have missed a literature entry), we would appreciate being contacted. The web site https://qmbench.net/ offers a place where such critically curated living databases can be made accessible in a convenient form.

## Supporting Information Available

Experimental details and infrared spectra; additional shift correlations and tables; details on the crowdedness index; and computational details (PDF)
