# Supplementary material for: Regularities and Anomalies in Neon Matrix Shifts of Hydrogen-Bonded O–H Stretching Fundamentals
Source: J Phys Chem A. 2024 Aug 19;128(34):7124–36. doi: 10.1021/acs.jpca.4c03468 (PMC11372756; doi:10.1021/acs.jpca.4c03468)
Supplement: Supplementary file 1 — jp4c03468_si_001.pdf [file jp4c03468_si_001.pdf]

# Supporting information: Regularities and anomalies in neon matrix shifts of hydrogen-bonded O-H stretching fundamentals

Margarethe Bödecker,[†] Dmytro Mihrin,[‡] Martin A. Suhm,[*,†] and René Wugt Larsen[*,‡]

†*Institute of Physical Chemistry, University of Göttingen, Tammannstrasse 6, 37077, Göttingen, Germany*

‡*Department of Chemistry, Technical University of Denmark, Kemitorvet 206, 2800 Kgs. Lyngby, Denmark*

E-mail: msuhm@gwdg.de; rewl@kemi.dtu.dk

## Contents

**List of Figures** **S2**

**List of Tables** **S3**

**1 Experiment** **S4**

1.1 Integration method . . . . . . . . . . . . . . . . . . . . . . . . . . . . . S4

1.2 Investigated compounds . . . . . . . . . . . . . . . . . . . . . . . . . . S7

1.3 FTIR spectra . . . . . . . . . . . . . . . . . . . . . . . . . . . . . . . . S8

**2 Supplementary neon matrix shift plots** **S17**

3 Crowdedness index S21

4 Harmonic modeling S22

4.1 Computational details . . . S22

4.2 xyz coordinates . . . S22

References S25

# List of Figures

S1 Integration limits . . . S4

S2 Acetonitrile−$H_2O$ and acrylonitrile−$H_2O$ jet spectra . . . S8

S3 Methylamine−$H_2O$ jet spectra . . . S9

S4 *i*-PrOH−$H_2O$ jet spectra . . . S10

S5 2-Aminoethanol Ne matrix spectra . . . S10

S6 *tert*-Butanol−methanol Ne matrix spectra . . . S11

S7 2-Propanol−$H_2O$ Ne matrix spectra . . . S11

S8 2,2,2-Trifluoroacetophenone−$H_2O$ Ne matrix spectra . . . S12

S9 Cyclobutanone−$H_2O$ Ne matrix spectra . . . S12

S10 Cycloheptanone−$H_2O$ Ne matrix spectra . . . S13

S11 Cyclooctanone−$H_2O$ Ne matrix spectra . . . S13

S12 Ethanol−$H_2O$ Ne matrix spectra . . . S14

S13 Formaldehyde−$H_2O$ Ne matrix spectra . . . S14

S14 Methanol−$H_2O$ Ne matrix spectra . . . S15

S15 *tert*-Butanol−$H_2O$ Ne matrix spectra . . . S15

S16 Tetrahydrofuran−$H_2O$ Ne matrix spectra . . . S16

S17 Tetrahydrothiophene−$H_2O$ Ne matrix spectra . . . S16

S18 Literature known Ne matrix shifts . . . S17

S19 Ne matrix shifts without deperturbation . . . S18
S20 Ne matrix shift plotted against complexation shift . . . S19

# List of Tables

S1 Integration limits . . . S5
S2 Ketone band positions and intensities . . . S5
S3 Uncertainties of virtual resonances . . . S6
S4 Used chemicals . . . S7
S5 Monomer jet and matrix wavenumbers . . . S19
S6 Full table of (deperturbed) data pairs . . . S20
S7 Van der Waals volumes and radii . . . S21
S8 Example structure for the determination of $d$ . . . S21
S9 Computational details . . . S22
S10 xyz coordinates of methylamine−water . . . S22
S11 xyz coordinates of methylamine−MeOH . . . S23
S12 xyz coordinates of dimethylamine−MeOH . . . S23
S13 xyz coordinates of ammonia−water . . . S24
S14 xyz coordinates of acetonitrile−water . . . S24
S15 xyz coordinates of acrylonitrile−water . . . S24
S16 xyz coordinates of pyridine−water . . . S25

# 1 Experiment

## 1.1 Integration method

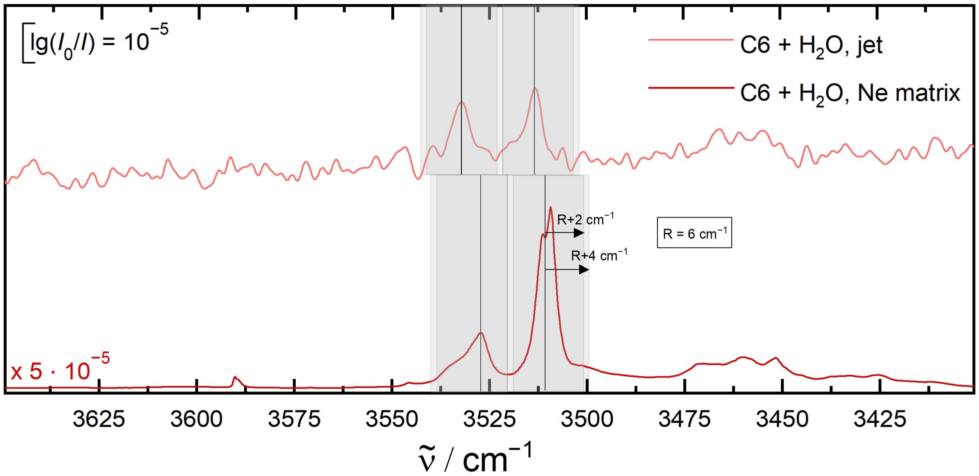


Figure S1: Exemplary visualization of the symmetric windows used for the integration of ketone hydrate signals in jet and matrix spectra. We identify the typical FWHM (full width at half maximum) of the signals and choose the larger of the integer wavenumber values among jet and matrix widths as a reference value $R$. We then integrate bands using a stochastic approach based on noise characteristics[1] around their estimated band center $B$ in the symmetric window $B \pm (R{+}2\,\mathrm{cm}^{-1})$ where $R$ is stochastically varied by $\pm U$ as a typical wing uncertainty together with synthetic noise to obtain statistical integral value distributions. This provides a first integral value $I_1$ with an uncertainty $\pm\Delta I_1$ (at 95% confidence interval). We repeat the integration in the larger window $B \pm (R{+}4\,\mathrm{cm}^{-1})$, again with stochastic variation by $\pm U$, to obtain another integral value $I_2$ with an uncertainty $\pm\Delta I_2$. The formulae for the determination of $I_1$ and $I_2$ are summarized in Table S1. We then apply maximum error propagation with the largest and smallest among the four values (typically, it will be $I_1 - \Delta I_1$ and $I_2 + \Delta I_2$ as long as the signals have wide wings which are only partially included in the integration because of their uncertain origin) for the resonance model treatment to obtain a range of possible intensity centers of the identified multiplet. Note that this stochastic integration procedure also provides a robust value for the center of intensity of asymmetric bands. For ketone hydrates, appropriate values chosen for the resonance integration are $R{=}6\,\mathrm{cm}^{-1}$ and $U{=}2\,\mathrm{cm}^{-1}$. Ideally, $R + U$ should not exceed the effective resonance coupling parameter $|W|$, which defines the minimum approach between two resonance partners $2W$. Otherwise, the signals of resonance partners may overlap and cannot be treated by separate integration. In this figure, the integration limits for $I_1$ and $I_2$ are marked in dark and light gray, respectively. C6 = cyclohexanone. For the jet spectrum, the resulting uncertainty ranges obtained for the two band integrals are (from right to left) 7-11 and 6-10 in units of $10^{-5}\,\mathrm{cm}^{-1}$ (largely limited by noise), whereas they are 4.2-4.5 and 1.1-1.3 $\mathrm{cm}^{-1}$ for the matrix spectrum (largely limited by overlapping weak absorption features), see Table S2.

Table S1: Formulae for the determination of the upper ($x_1$) and lower ($x_2$) integration limit for signal integration methods $I_{1,2}$. $B$ = position of band center in $cm^{-1}$, $R$ = full width at half maximum of the broadest peak in spectra of this compound class in $cm^{-1}$.

| | $x_1$ | $x_2$ |
|---|---|---|
| $I_1$ | $B + (R + 2\,cm^{-1}) \pm 2\,cm^{-1}$ | $B - (R + 2\,cm^{-1}) \pm 2\,cm^{-1}$ |
| $I_2$ | $B + (R + 4\,cm^{-1}) \pm 2\,cm^{-1}$ | $B - (R + 4\,cm^{-1}) \pm 2\,cm^{-1}$ |
| example: cyclohexanone + $H_2O$, jet, $B = 3532\,cm^{-1}$, $R = 6\,cm^{-1}$ | | |
| $I_1$ | $(3540 \pm 2)\,cm^{-1}$ | $(3524 \pm 2)\,cm^{-1}$ |
| $I_2$ | $(3542 \pm 2)\,cm^{-1}$ | $(3522 \pm 2)\,cm^{-1}$ |

Table S2: Band positions ($B_I$, $B_{II}$) and intensities ($I_I$, $I_{II}$) of the monohydrate signals of cyclohexanone, acetophenone and acetone. The integration limits were determined using the formulae listed in Table S1. $R = 6\,cm^{-1}$.

| acceptor | experiment | $B_I/cm^{-1}$ | $I_I$ | $B_{II}/cm^{-1}$ | $I_{II}$ |
|---|---|---|---|---|---|
| cyclohexanone | jet | 3514 | $I_1$: $(7.9\pm0.9)\cdot10^{-5}$ | 3532 | $I_1$: $(6.7\pm0.9)\cdot10^{-5}$ |
| | jet | 3514 | $I_2$: $(10.0\pm1.1)\cdot10^{-5}$ | 3533 | $I_2$: $(9.3\pm1.1)\cdot10^{-5}$ |
| | Ne matrix | 3510 | $I_1$: $4.24\pm0.02$ | 3528 | $I_1$: $1.14\pm0.02$ |
| | Ne matrix | 3510 | $I_2$: $4.490\pm0.005$ | 3530 | $I_2$: $1.27\pm0.02$ |
| acetophenone | jet | 3515 | $I_1$: $(8.6\pm0.9)\cdot10^{-5}$ | 3536 | $I_1$: $(32.0\pm0.9)\cdot10^{-5}$ |
| | jet | 3515 | $I_2$: $(11.1\pm1.0)\cdot10^{-5}$ | 3536 | $I_2$: $(33.8\pm1.1)\cdot10^{-5}$ |
| | Ne matrix | 3513 | $I_1$: $7.31\pm0.02$ | 3531 | $I_1$: $6.16\pm0.03$ |
| | Ne matrix | 3513 | $I_2$: $7.620\pm0.006$ | 3531 | $I_2$: $6.840\pm0.009$ |
| acetone | jet | 3515 | $I_1$: $(17.5\pm0.9)\cdot10^{-5}$ | 3537 | $I_1$: $(46.1\pm0.9)\cdot10^{-5}$ |
| | jet | 3515 | $I_2$: $(18.3\pm1.0)\cdot10^{-5}$ | 3538 | $I_2$: $(52.7\pm1.1)\cdot10^{-5}$ |
| | Ne matrix | 3515 | $I_1$: $4.900\pm0.007$ | 3533 | $I_1$: $1.570\pm0.007$ |
| | Ne matrix | 3515 | $I_2$: $5.010\pm0.003$ | 3533 | $I_2$: $1.49\pm0.02$ |

Table S3: Monohydrate systems, where no resonance was observed in the jet, but resonance signals could potentially hide below the detection limit. $B$ = band position; $W$ = potential size of a coupling matrix element; $S/N$ = signal-to-noise ratio, determined by dividing the amplitude of the $OH_b$ signal by the noise amplitude; $\Delta B$ = estimated uncertainty of the band position due to hidden resonances. $\Delta B = \left| B - \frac{(S/N)\cdot B + 1\cdot(B\pm 2W)}{(S/N)+1} \right| = \frac{2W}{(S/N)+1}$ is obtained by assuming a single virtual resonance band with $S/N = 1$ at $B \pm 2W$. For all $OH_b$ modes which are experimentally observed below $3550\,\mathrm{cm^{-1}}$, $W = 10\,\mathrm{cm^{-1}}$ [3] for the coupling between $OH_b$ and b2lib is implied. For $B < 3450\,\mathrm{cm^{-1}}$, a larger $OH_b$−b2ON matrix coupling element of $W = 30\,\mathrm{cm^{-1}}$ [7] is implied.

| acceptor | $B/\mathrm{cm^{-1}}$ | $W/\mathrm{cm^{-1}}$ | $S/N$ | $\Delta B/\mathrm{cm^{-1}}$ |
|---|---|---|---|---|
| tetrahydrothiophene[2] | 3507 | 10 | 8 | 3 |
| cyclooctanone[2] | 3503 | 10 | 25 | 1 |
| cyclobutanone[3] | 3548 | 10 | 8 | 3 |
| ethanol[4] | 3548 | 10 | 13 | 2 |
| oxirane[5] | 3542 | 10 | 23 | 1 |
| 2-propanol[TW] | 3537 | 10 | 25 | 1 |
| *t*-butanol[6] | 3530 | 10 | 10 | 2 |
| tetrahydrofuran[2] | 3491 | 10 | 8 | 3 |
| ammonia[TW] | 3486 | 10 | 5 | 4 |
| pyridine[2] | 3454 | 10 | 11 | 2 |
| methylamine[TW] | 3417 | 30 | 7 | 8 |

## 1.2 Investigated compounds

Table S4: Names, formulae, CAS numbers, suppliers, and purities of used chemicals in the combined jet and neon matrix experiments. For the jet measurements, the substances were purified by a *freeze-pump-thaw*-cycle (as described in ref. 8), Acrylonitrile was protected from light to prevent polymerization and the aqueous solutions of ammonia, methylamine, and dimethylamine were refilled at least after every second measurement so that a sufficient concentration of the solute in the solution was secured.

| name | formula | CAS number | supplier | purity |
|---|---|---|---|---|
| helium | He | 7440-59-7 | Nippon | 99.996 % |
| neon | Ne | 7440-01-9 | Linde | 99.996 % |
| water | $H_2O$ | 7732-18-5 | - | demineralized |
| | $^{18}OH_2$ | 14314-42-2 | Sigma Aldrich | 97% |
| methanol | $CH_3OH$ | 67-56-1 | TCI chemicals | 99.8% |
| 2-propanol | $(CH_3)_2CHOH$ | 67-63-0 | TCI chemicals | 99.5% |
| ammonia | $NH_3$ | 7664-41-7 | Sigma Aldrich | 30-33% in $H_2O$ |
| methylamine | $CH_3NH_2$ | 74-89-5 | Sigma Aldrich | 40% in $H_2O$ |
| dimethylamine | $(CH_3)_2NH$ | 124-40-3 | Sigma Aldrich | 40% in $H_2O$ |
| acetonitrile | $CH_3CN$ | 75-05-8 | TCI chemicals | 99.5% |
| acrylonitrile | $C_2H_3CN$ | 107-13-1 | TCI chemicals | 99%, MEHQ stab. |
| 2,2,2-trifluoro-acetophenone | $CF_3COC_6H_5$ | 434-45-7 | Sigma Aldrich | 99% |
| 2-aminoethanol | $NH_2(CH_2)_2OH$ | 141-43-5 | Sigma Aldrich | 99% |
| acetone | $CH_3COCH_3$ | 67-64-1 | Sigma Aldrich | 99.9% |
| acetophenone | $CH_3COC_6H_5$ | 98-86-2 | Sigma Aldrich | 99% |
| cyclobutanone | $C_4H_6(=O)$ | 1191-95-3 | Sigma Aldrich | 99% |
| cyclohexanone | $C_6H_{10}(=O)$ | 108-94-1 | Sigma Aldrich | 99.8% |
| cycloheptanone | $C_7H_{12}(=O)$ | 502-42-1 | Sigma Aldrich | 99% |
| cyclooctanone | $C_8H_{14}(=O)$ | 502-49-8 | Sigma Aldrich | 98% |
| deuterium oxide | $D_2O$ | 7789-20-0 | Sigma Aldrich | 99% D-atoms |
| ethanol | $CH_3CH_2OH$ | 64-17-5 | Sigma Aldrich | 99.9% |
| paraformaldehyde | $HO(CH_2O)_nH$ | 30525-89-4 | Sigma Aldrich | 95% |
| phenol | $C_6H_5OH$ | 108-95-2 | Sigma Aldrich | 99% |
| pyridine | $C_5H_5N$ | 110-86-1 | Sigma Aldrich | 99% |
| *tert*-butanol | $(CH_3)_3COH$ | 75-65-0 | Sigma Aldrich | 99.8%, anhydrous |
| tetrahydrofuran | $C_4H_8O$ | 109-99-9 | Sigma Aldrich | 99.9%, inh.-free |
| tetrahydrothiophene | $C_4H_8S$ | 110-01-0 | Sigma Aldrich | 99% |

## 1.3 FTIR spectra

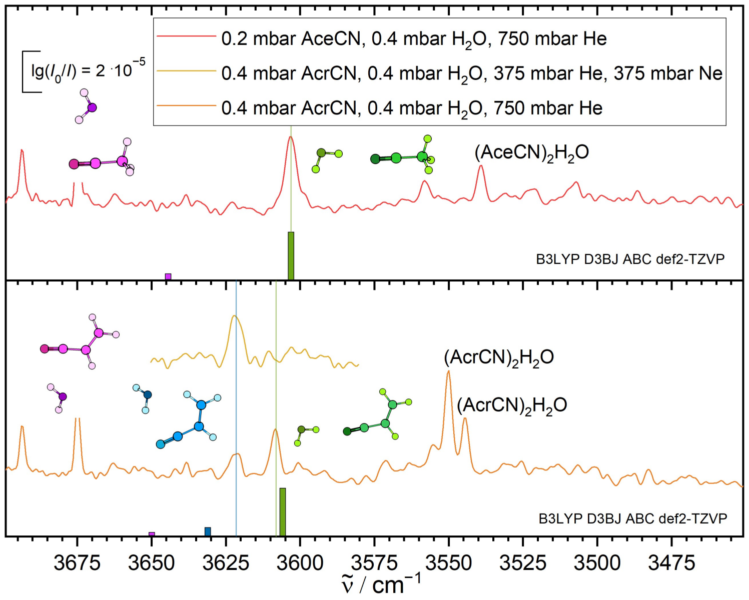


Figure S2: FTIR jet spectra of the OH stretching region of water-nitrile clusters. While for the water−acetonitrile (AceCN, top) 1:1 complex only one conformer was observed, two conformers are observed for the 1:1 complex of water−acrylonitrile (AcrCN, bottom) in He as a carrier gas. The more stable one is identified by admixture of neon to the carrier gas. The bars below the spectra indicate relative harmonic IR intensity and wavenumber predictions, the latter wavenumber-scaled by 0.9702 to match the single AceCN band for assignment guidance. The purple-colored conformers obtained from calculations were not observed in the jet spectra.

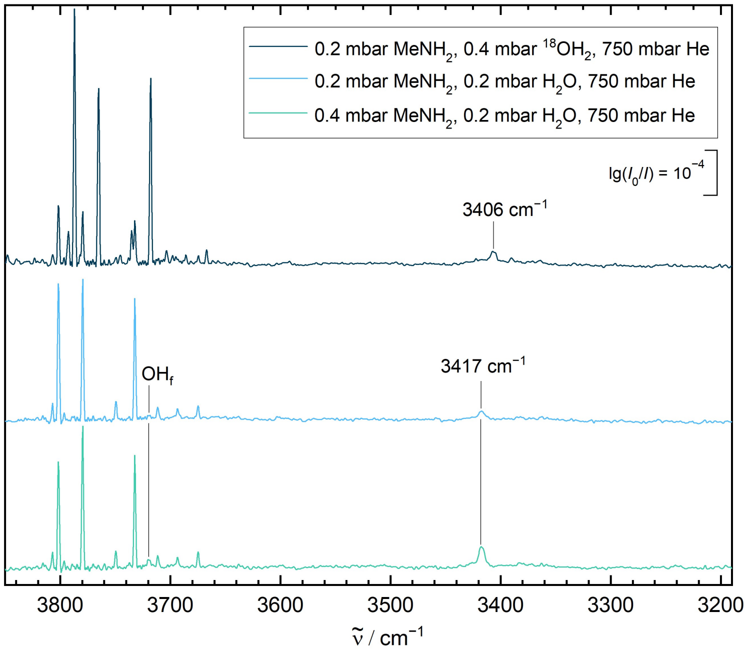


Figure S3: FTIR jet spectra of methylamine and water with helium as carrier gas, recorded for two different partial pressures of methylamine. Apart from the water monomer signals above 3650 $\mathrm{cm^{-1}}$ and a free OH stretching fundamental near 3720 $\mathrm{cm^{-1}}$, the spectrum features only one signal at 3417 $\mathrm{cm^{-1}}$ above the noise level, which is assigned to the methylamine monohydrate. $^{18}$O substitution shows that the transition is centered on the water unit and does not correspond to a hydrogen bond-intensified NH stretching vibration of the amine. An improved S/N ratio will be required to rule out or identify weaker resonance contributions to the signal.

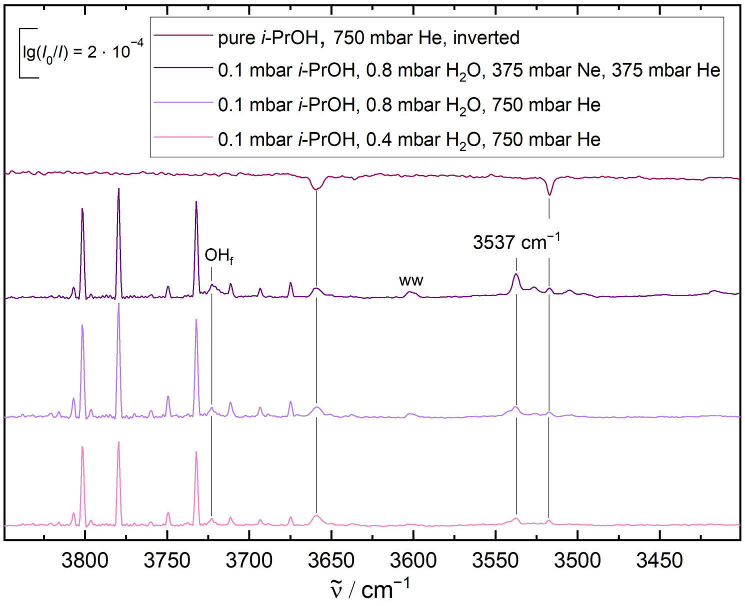


Figure S4: FTIR jet spectra of *iso*-propanol (2-propanol) and water (w) at different water concentrations for varying carrier gas mixtures. The global minimum structure of the *i*-PrOH monohydrate is assigned at 3537 $cm^{-1}$.

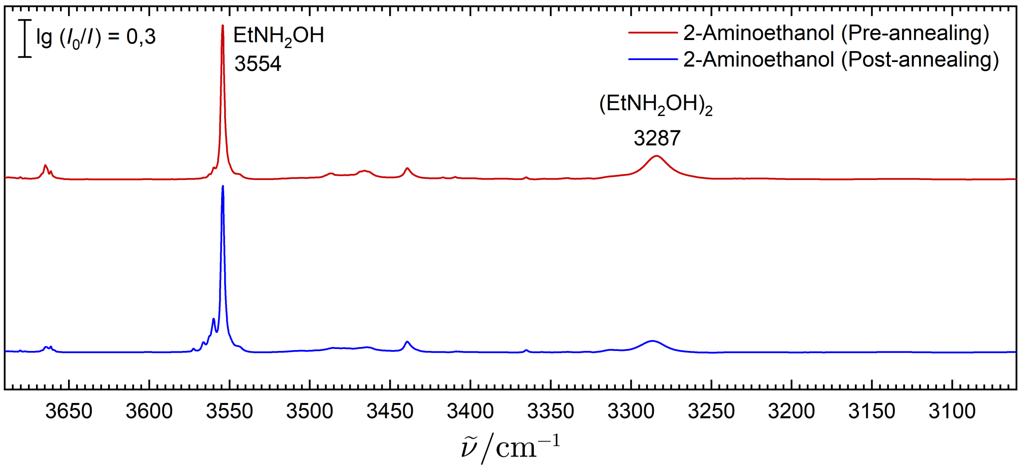


Figure S5: Pre- and post-annealing neon matrix spectra of 2-aminoethanol.

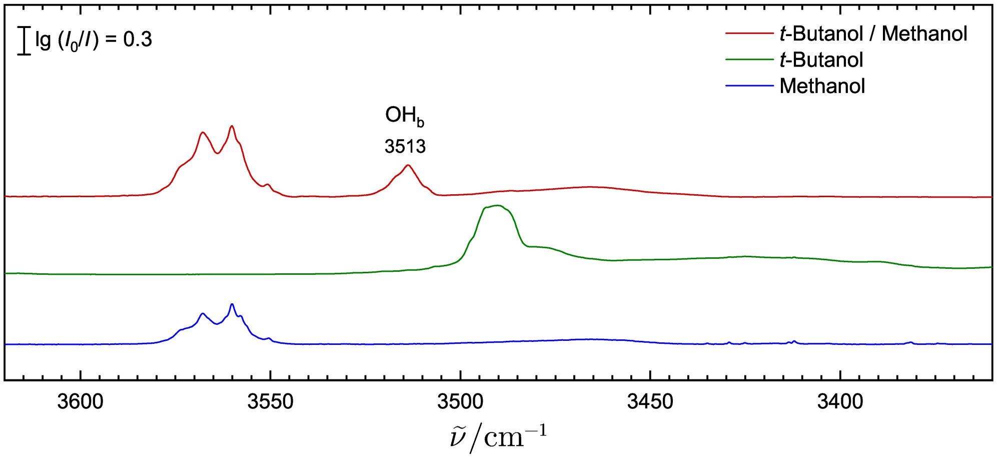


Figure S6: Neon matrix spectra of pure methanol (blue), pure *tert*-butanol (green) and the mixed spectrum (red).

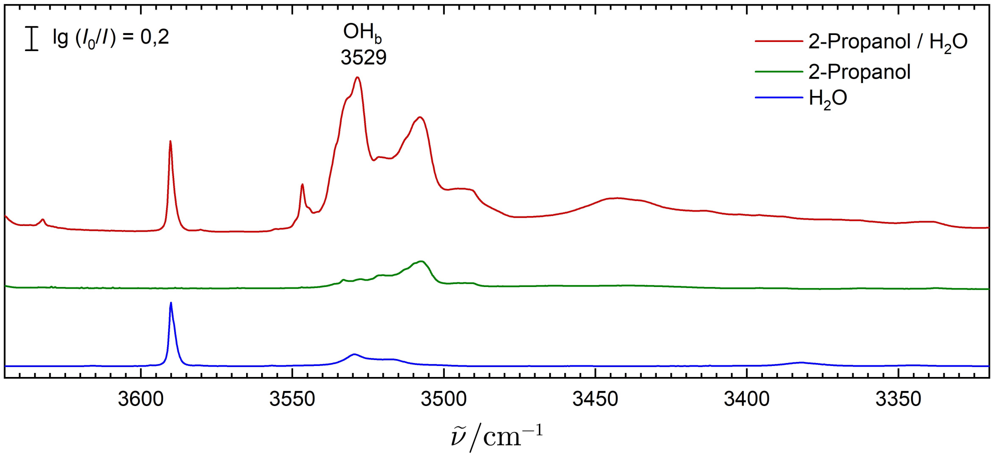


Figure S7: Neon matrix spectra of pure water (blue), pure 2-propanol (green) and the mixed spectrum (red). For the 2-propanol monohydrate, a previous Ne matrix study[9] rules out conformational switching.

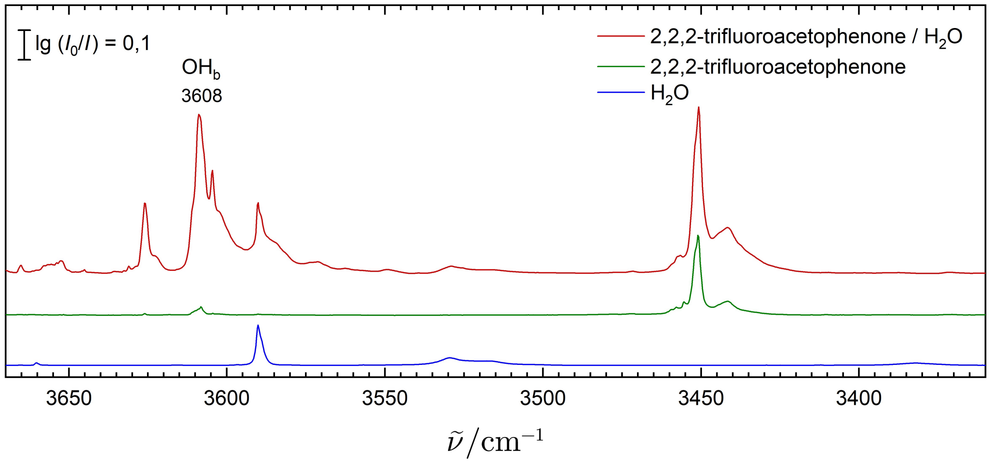


Figure S8: Neon matrix spectra of pure water (blue), pure 2,2,2-trifluoroacetophenone (green) and the mixed spectrum (red). The band near 3625 $cm^{-1}$ is also believed to be due to a hydrate complex.

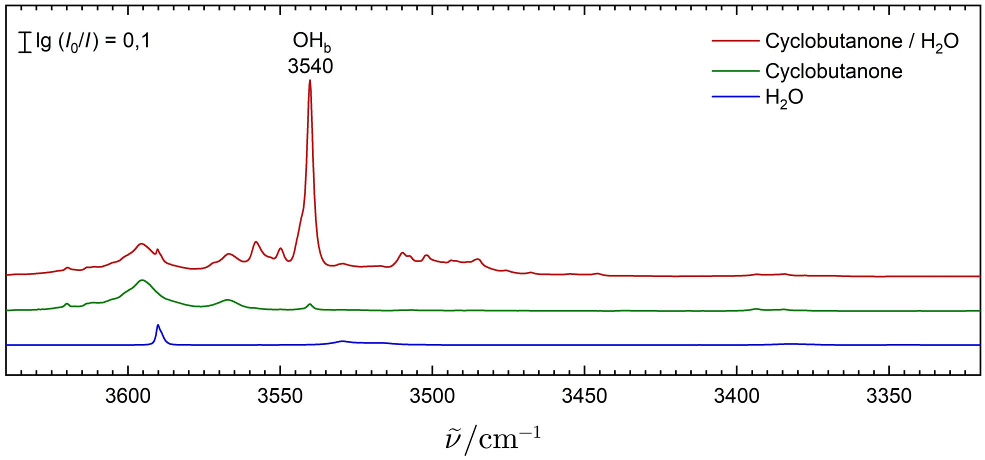


Figure S9: Neon matrix spectra of pure water (blue), pure cyclobutanone (green) and the mixed spectrum (red).

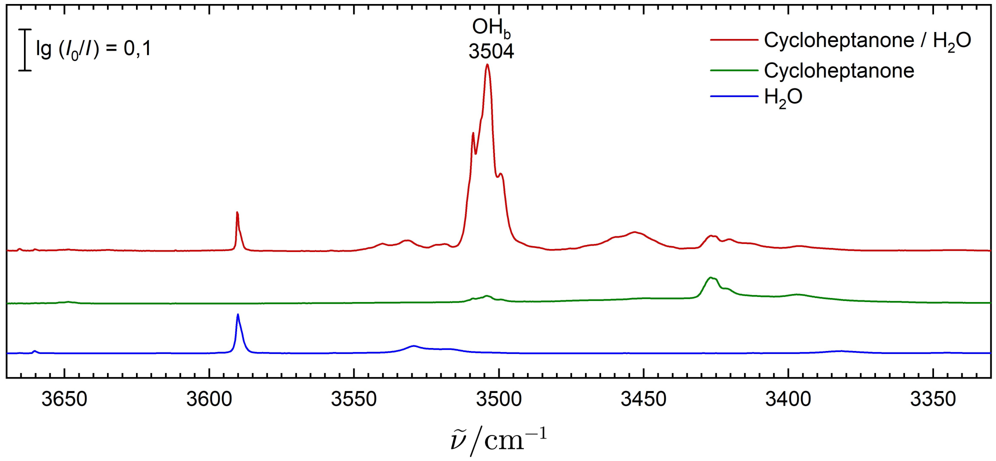


Figure S10: Neon matrix spectra of pure water (blue), pure cycloheptanone (green) and the mixed spectrum (red). According to quantum chemical calculations (B3LYP-D3(BJ,abc)/def2-TZVP, see Table S9) a spectral and energetic overlap between two nearly degenerate conformations (zero-point corrected energy difference $\Delta E_0 = 0.5\,\mathrm{kJ\,mol^{-1}}$; $\Delta\tilde{\nu}_{\mathrm{OHb}} = 3\,\mathrm{cm^{-1}}$) is conceivable. A hypothetical conformational switch induced by the matrix would thus only have a minor influence on the derived matrix shift.

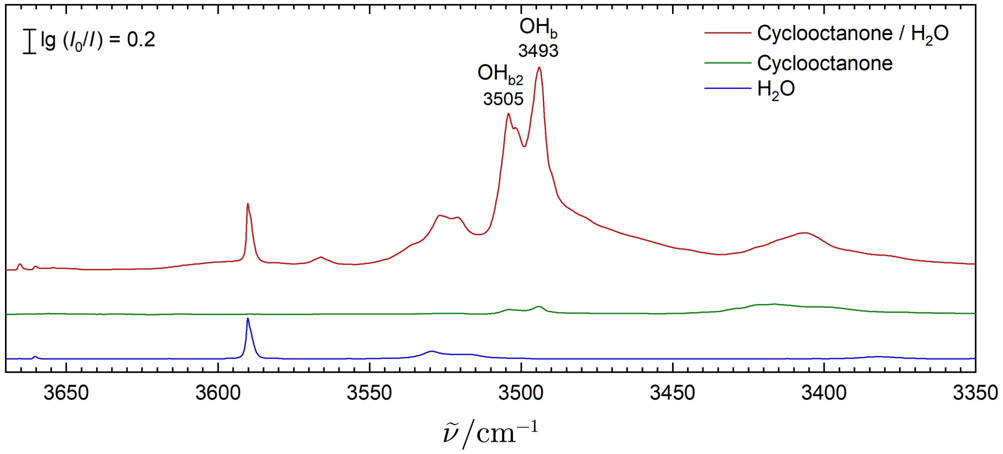


Figure S11: Neon matrix spectra of pure water (blue), pure cyclooctanone (green) and the mixed spectrum (red). The monohydrate isomerism (major conformation OH$_b$, minor conformation OH$_{b2}$) seems to be robustly reflected in microwave[10], infrared jet[2] and infrared matrix spectra, with the major monohydrate conformation having the lower OH$_b$ wavenumber in both the jet and the matrix infrared spectra. There is no evidence for a conformational switch induced by the matrix, although the predicted energy difference is quite subtle ($\Delta E_0 = 0.5\,\mathrm{kJ\,mol^{-1}}$ at B3LYP-D3(BJ,abc)/def2-TZVP level of theory).

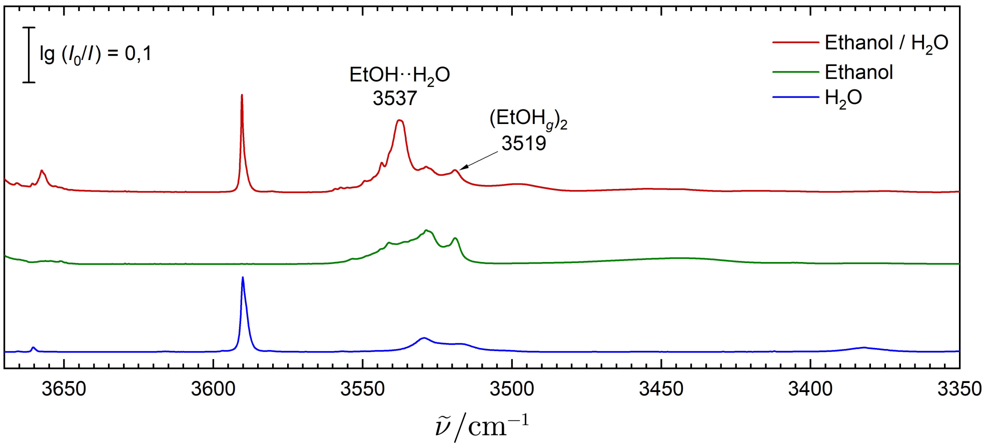


Figure S12: Neon matrix spectra of pure water (blue), pure ethanol (green) and the mixed spectrum (red). Concerning the ethanol homodimer, the analogy between the Ne matrix and the jet[11] suggests a corresponding isomer assignment. In the case of w−ethanol, a previous Ne matrix study[12] rules out conformational switching.

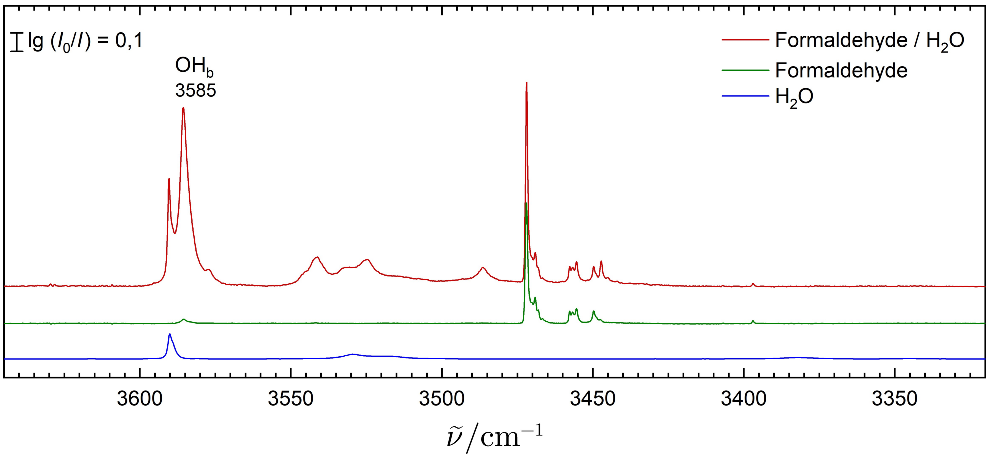


Figure S13: Neon matrix spectra of pure water (blue), pure formaldehyde (green) and the mixed spectrum (red).

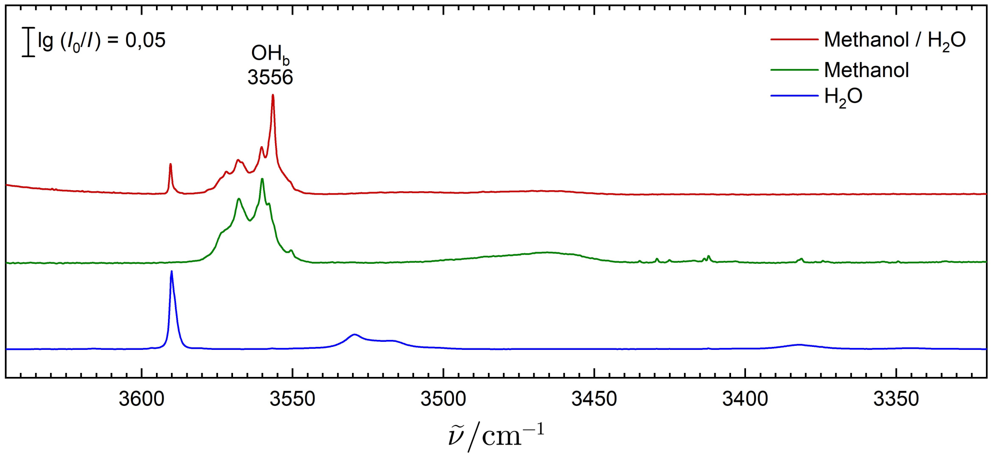


Figure S14: Neon matrix spectra of pure water (blue), pure methanol (green) and the mixed spectrum (red).

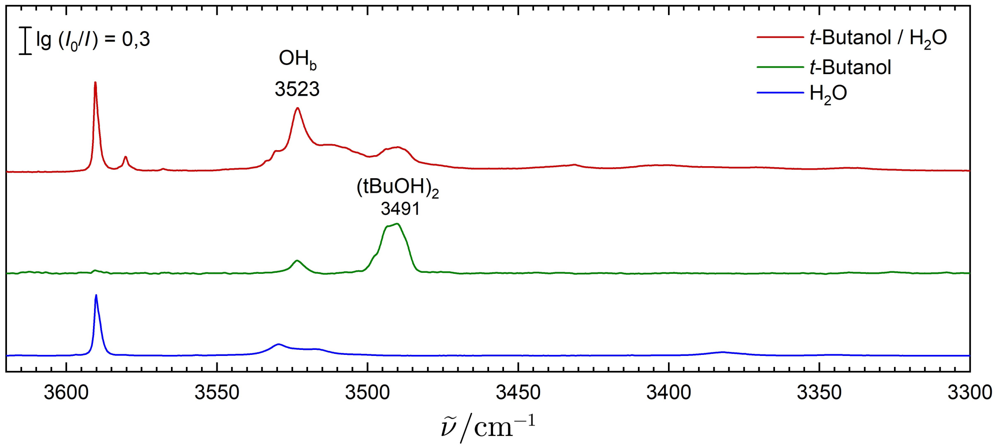


Figure S15: Neon matrix spectra of pure water (blue), pure *tert*-butanol (green) and the mixed spectrum (red).

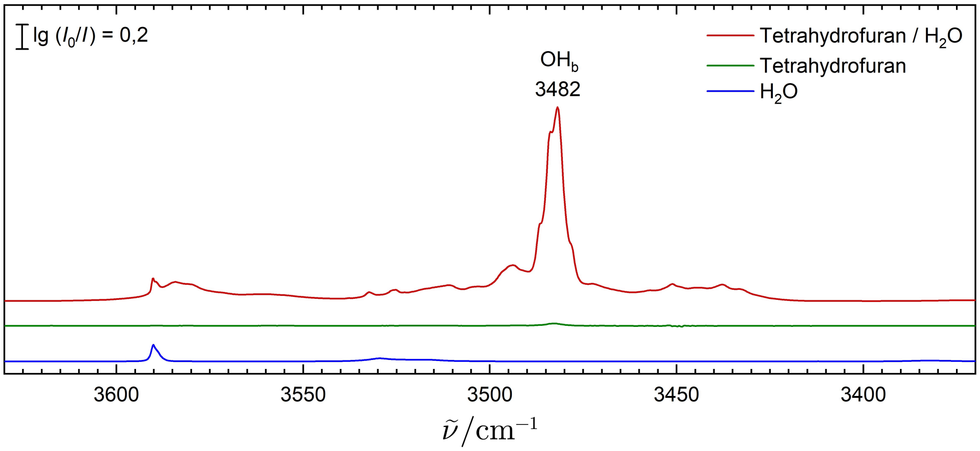


Figure S16: Neon matrix spectra of pure water (blue), pure tetrahydrofuran (green) and the mixed spectrum (red).

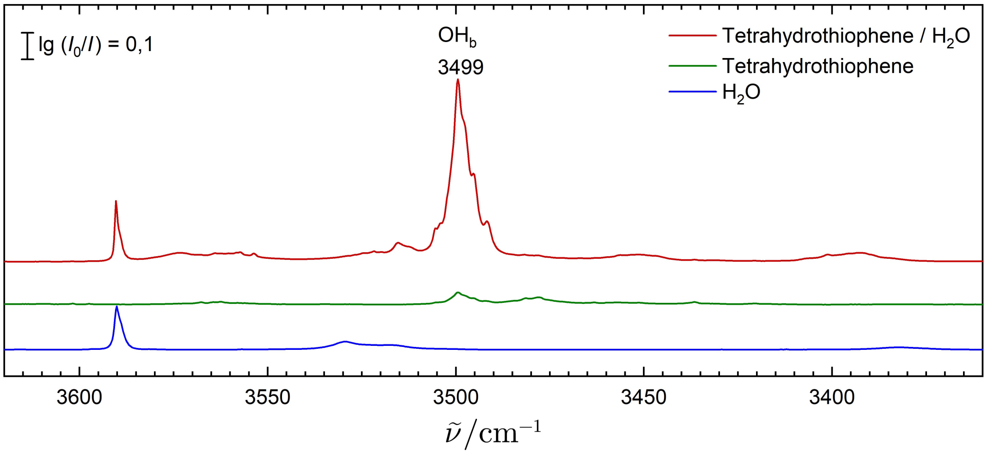


Figure S17: Neon matrix spectra of pure water (blue), pure tetrahydrothiophene (green) and the mixed spectrum (red).

## 2 Supplementary neon matrix shift plots

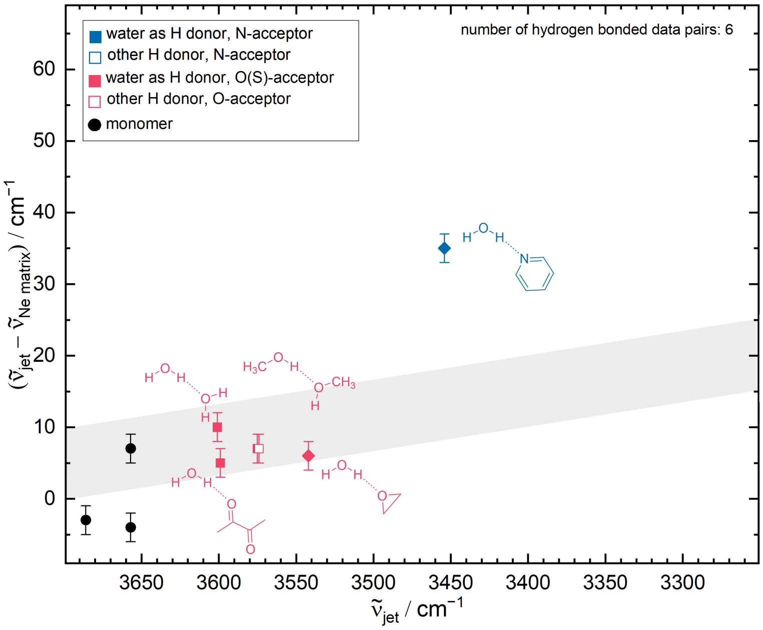


Figure S18: Literature known Ne matrix shifts plotted against the wavenumber of the strongest hydrogen bonded OH stretching jet signal for 6 dimers (2x diacetyl−water, methanol−methanol, oxirane−water, water−water and pyridine−water) and in black for 3 free monomers (water, methanol and phenol). The symbols of the data points and/or error bars that are (potentially) affected by resonances in the jet and/or matrix spectra are tilted by 45°. The systems concerned are shown in Table 3 in the main document.

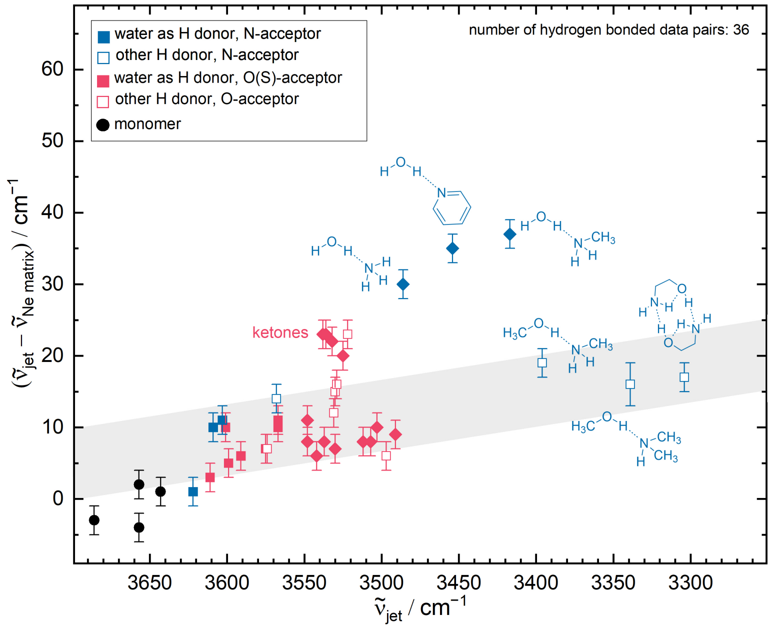


Figure S19: Ne matrix shifts without deperturbation of resonances plotted against the wavenumber of the strongest hydrogen bonded OH stretching jet signal for 36 dimers or internally hydrogen bonded monomers (and in black for 4 free monomers which act as donors in the dimers). The symbols of the data points and/or error bars that are (potentially) affected by resonances in the jet and/or matrix spectra are tilted by 45°. The systems concerned are shown in Table 3 in the main document. Without deperturbation, some ketone and amine monohydrates show anomalously large Ne matrix shifts.

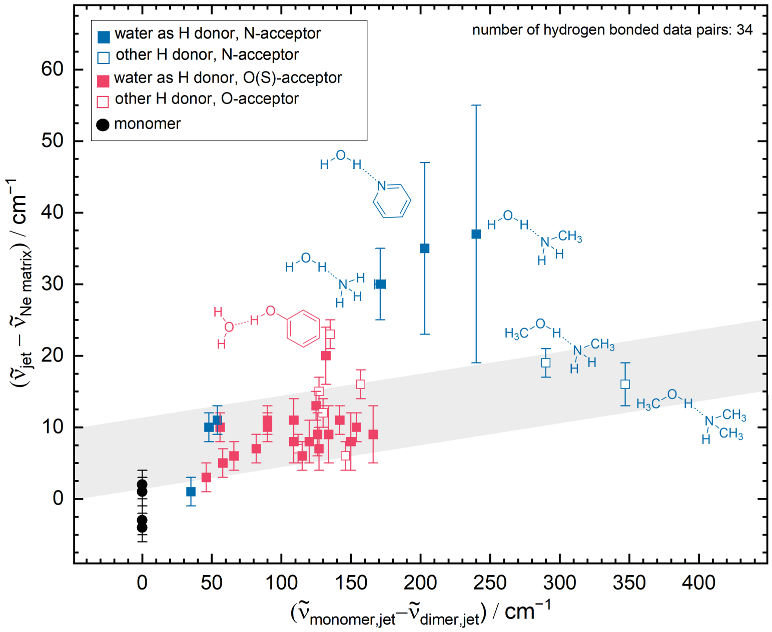


Figure S20: Deperturbed Ne matrix shifts plotted against the jet complexation shift wavenumber for 34 dimers (and in black for 4 free monomers which act as donors in the dimers).

Table S5: Monomer wavenumbers (in cm$^{-1}$) for the OH donors in the complexes of Table 1 and 2 (main document) in the gas phase and in the neon matrix together with the resulting matrix downshifts $\Delta$. In contrast to 1:1 complexes, the sign of the Ne matrix shifts is non-uniform (negative values correspond to wavenumber upshifts). $^{TW}$=This work.

| Donor | $\tilde{\nu}_{jet}$ | $\tilde{\nu}_{mat}$ | $\Delta$ |
|---|---|---|---|
| w | 3657 [13] | 3661 [14] | −4 |
| methanol | 3686 [15] | 3689 [16], 3690 [17] | −3, −4 |
| phenol | 3657 [18] | (3650 [19]), 3655$^{TW}$ | (+7), +2$^{TW}$ |
| *tert*-butanol | 3643 [20] | 3642$^{TW}$ | +1 |

Table S6: Data pairs used in Figure 1: (Deperturbed) wavenumber downshifts $\Delta$ of hydrogen-bonded O-H stretching vibrations in the Ne matrix compared to the cold gas phase values for different OH−O(S) and OH−N donor−acceptor pairs using FTIR techniques. Resonance-cases and non-resonance cases are shown. All numbers in $cm^{-1}$. $^{TW}$=This work. w=$H_2O$. Sorted by decreasing wavenumber but keeping isomers (*) together.

| | $\tilde{\nu}_{jet}$ | $\tilde{\nu}_{mat}$ | $\Delta$ |
|---|---|---|---|
| OH−O(S) Species | | | |
| w−2,2,2-trifluoroacetophenone | 3611(1)[2] | 3608(1)[TW] | 3(2) |
| w−w | 3601(1)[21] | 3591[22] | 10(2) |
| w−diacetyl | 3599(1)[23] | 3594(1)[23] | 5(2) |
| w−diacetyl (*) | 3575(1)[23] | 3568(1)[23] | 7(2) |
| w−formaldehyde | 3591(1)[2] | 3585(1)[TW] | 6(2) |
| methanol−methanol | 3574(1)[24] | 3567(1)[25] | 7(2) |
| w−methanol | 3567(1)[4] | 3556(1)[TW] | 11(2) |
| w−cyclobutanone | 3548(2)[3] | 3540(1)[TW] | 8(3) |
| w−ethanol | 3548(2)[4] | 3537(1)[TW] | 11(3) |
| w−oxirane | 3542(1)[5] | 3536(1)[5] | 6(2) |
| w−2-propanol | 3537(2)[TW] | 3529(1)[TW] | 8(3) |
| w−acetone | 3532(1)[3] | 3519(1)[TW] | 13(2) |
| w−acetophenone | 3531(2)[3] | 3522(1)[TW] | 9(3) |
| w−acetophenone (*) | 3567(1)[3] | 3557(1)[TW] | 10(2) |
| w−*t*-butanol | 3530(2)[6] | 3523(1)[TW] | 7(3) |
| w−cyclohexanone | 3523(3)[3] | 3514(1)[TW] | 9(4) |
| ethanol (g)−ethanol (g) | 3531(1)[11] | 3519(1)[TW] | 12(2) |
| phenol−phenol | 3530(1)[26] | 3515(1)[TW] | 15(2) |
| methanol−*t*-butanol | 3529(1)[20] | 3513(1)[TW] | 16(2) |
| phenol−w | 3522(1)[27,28] | 3499(1)[TW] | 23(2) |
| w−cycloheptanone | 3515(1)[3] | 3504(1)[TW] | 11(2) |
| w−tetrahydrothiophene | 3507(3)[2] | 3499(1)[TW] | 8(4) |
| w−cyclooctanone | 3503(1)[2] | 3493(1)[TW] | 10(2) |
| w−cyclooctanone (*) | 3525(3)[2] | 3505(1)[TW] | 20(4) |
| *t*-butanol−*t*-butanol | 3497(1)[20] | 3491(1)[TW] | 6(2) |
| w−tetrahydrofuran | 3491(3)[2] | 3482(1)[TW] | 9(4) |
| OH−N Species | | | |
| w−acrylonitrile | 3622(1)[TW] | 3621(1)[29] | 1(2) |
| w−acrylonitrile (*) | 3609(1)[TW] | 3599(1)[29] | 10(2) |
| w−acetonitrile | 3603(1)[TW] | 3592(1)[22] | 11(2) |
| 2-aminoethanol (M) | 3568(1)[30] | 3554(1)[TW] | 14(2) |
| w−ammonia | 3486(4)[TW] | 3456(1)[31] | 30(5) |
| w−pyridine | 3454(2)[2] | 3419(10)[32] | 35(12) |
| w−methylamine | 3417(8)[TW] | 3380(10)[33] | 37(18) |
| methanol−methylamine | 3396(1)[TW] | 3377(1)[TW] | 19(2) |
| methanol−dimethylamine | 3339(1)[TW] | 3323(2)[TW] | 16(3) |
| 2-aminoethanol−2-aminoethanol | 3304(1)[30] | 3287(1)[TW] | 17(2) |

# 3 Crowdedness index

Table S7: Van der Waals volumes $V$ and radii $r$ of some structural units according to Ref. 34, used to calculate the crowdedness index.

| structural unit | $V$ / cm$^3$ mol$^{-1}$ | $r$ / Å |
|---|---|---|
| $-CH_3$ | 13.67 | 1.756 |
| $-CH_2-$ | 10.23 | 1.595 |
| $-C\equiv$ | 8.05 | 1.47 |
| $>CH-$ | 6.78 | 1.39 |
| $>C<$ | 3.33 | 1.10 |
| $-H$ | 3.00 | 1.06 |

Table S8: The distances $d_i$ between the structural units and the hydrogen bonding H atom, shown for the example of dimethylamine–methanol. $r_i$ is the van der Waals radius of the structural unit shown in Table S7. In this case, the number of structural units (excluding the N and the O atom) included in the summation of the index $C$ is $N = 4$. All structures were optimized at B3LYP-D3(BJ,abc)/def2-TZVP level using ORCA[35] (for details, see Table S9).

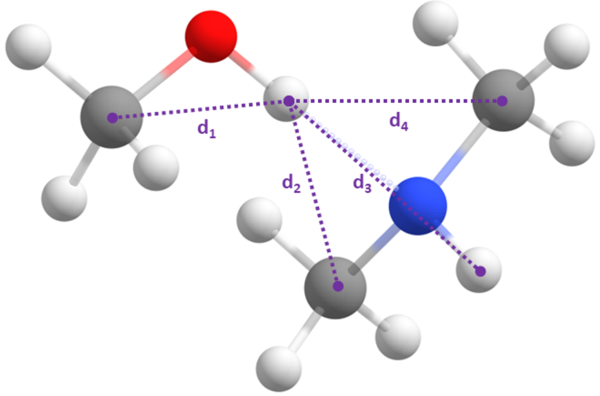


| $i$ | structural unit | $d_i$ / Å | $r_i$ / Å |
|---|---|---|---|
| 1 | $-CH_3$ | 1.86 | 1.756 |
| 2 | $-CH_3$ | 2.64 | 1.756 |
| 3 | $-H$ | 2.50 | 1.06 |
| 4 | $-CH_3$ | 2.61 | 1.756 |

# 4 Harmonic modeling

## 4.1 Computational details

Table S9: The lowest energy dimers were obtained using Crest[36,37] at GFN2-xTB[38] level and then reoptimized at B3LYP-D3(BJ,abc)/def2-TZVP level using Orca[35] (version 5.0.4). Orca keywords are given in the table.

| method | keywords |
|---|---|
| B3LYP-D3(BJ,abc)/def2-TZVP | defgrid3, verytightscf, verytightopt, freq |

## 4.2 xyz coordinates

Table S10: xyz coordinates of the methylamine−water structure in Å optimized at B3LYP-D3(BJ,abc)/def2-TZVP level using Orca 5.0.4.

| atom | x | y | z |
|---|---|---|---|
| C | −1.49006871373909 | −0.53816852703606 | 0.00364989204715 |
| H | −1.30444157131306 | −1.04407638936628 | −0.94381068024555 |
| H | −2.57328923387025 | −0.45187591836961 | 0.14670184619556 |
| H | −1.08419028187113 | −1.16991432188195 | 0.79371292490590 |
| N | −0.77767072056971 | 0.74637684197528 | 0.00680096923713 |
| H | −0.92461213290321 | 1.23763114417896 | 0.88098755782362 |
| H | −1.11570570988006 | 1.34857482307317 | −0.73466279361242 |
| H | 1.03178510794288 | 0.13645913175473 | −0.13786891842464 |
| O | 1.86521611028626 | −0.37918355526443 | −0.16405687816558 |
| H | 2.55800714591736 | 0.25491677093620 | −0.36993391976116 |

Table S11: xyz coordinates of the methylamine−MeOH structure in Å optimized at B3LYP-D3(BJ,abc)/def2-TZVP level using Orca 5.0.4.

| atom | x | y | z |
|---|---|---|---|
| C | 2.02182217013845 | 0.55471865667403 | −0.28797411330224 |
| H | 1.52945410147067 | 1.52939828651908 | −0.41237420178780 |
| H | 2.19486799775548 | 0.13090770958189 | −1.28661722742266 |
| H | 2.99411928730816 | 0.72824562750304 | 0.17582666539285 |
| O | 1.29184963694565 | −0.31582504488407 | 0.54843013968525 |
| H | 0.41152203174801 | −0.46410646490730 | 0.14495669873198 |
| H | −1.82641723208987 | 0.38486165137107 | 1.39847182893755 |
| C | −2.04927931105487 | 0.52742934376466 | 0.34111819954529 |
| H | −3.13719786154065 | 0.57319026963689 | 0.21678073021191 |
| N | −1.39811674566390 | −0.53700674165249 | −0.43201798563336 |
| H | −1.77024884812548 | −1.44586656645980 | −0.18181884083579 |
| H | −1.55569441153023 | −0.41426961001448 | −1.42535098301143 |
| H | −1.62896081536143 | 1.4893328828674H8 | 0.04647908948845 |

Table S12: xyz coordinates of the dimethylamine−MeOH structure in Å optimized at B3LYP-D3(BJ,abc)/def2-TZVP level using Orca 5.0.4.

| atom | x | y | z |
|---|---|---|---|
| N | −0.95577167064816 | 0.09539567207503 | 0.65524129004215 |
| H | −0.98540251193691 | 0.32314254698140 | 1.64161582115225 |
| C | −1.33704510379557 | 1.26036664977882 | −0.13733429841552 |
| H | −2.39569969653738 | 1.53505569538990 | −0.02465791751956 |
| H | −0.72227952814917 | 2.11463657898720 | 0.14734040092001 |
| H | −1.15196003269904 | 1.04911492327077 | −1.19217137610530 |
| C | −1.78751548428073 | −1.07533875246747 | 0.39020859094809 |
| H | −2.86149572940406 | −0.88223650071740 | 0.52448172090417 |
| H | −1.62628316973356 | −1.40101097617839 | −0.63902956700616 |
| H | −1.49134122965177 | −1.89124035133798 | 1.04947066519995 |
| H | 2.12628514749616 | 1.29075884222423 | −0.84697405652704 |
| C | 2.46893085617672 | 0.31718005641011 | −0.46958709547162 |
| H | 2.81202028870913 | 0.45425785513859 | 0.56484861517551 |
| H | 3.32661766721918 | 0.00860754930907 | −1.06940460635475 |
| O | 1.47914594874263 | −0.68236444248035 | −0.58099787341454 |
| H | 0.70402424849252 | −0.41314534638355 | −0.04246031352764 |

Table S13: xyz coordinates of the ammonia−water structure in Å optimized at B3LYP-D3(BJ,abc)/def2-TZVP level using Orca 5.0.4.

| atom | x | y | z |
|---|---|---|---|
| N | 1.46220922387501 | −0.00796167818213 | −0.00073311721058 |
| H | 1.93934641559128 | −0.61985119840353 | −0.65301025912248 |
| H | 1.85232575538069 | −0.17217131615211 | 0.92051095400343 |
| H | 1.68123160397369 | 0.94763456393714 | −0.26040620128827 |
| H | −0.49843097711843 | −0.03952105090298 | 0.01282104804513 |
| O | −1.47014587480114 | 0.06294821646304 | 0.03272632883267 |
| H | −1.82479614690111 | −0.82768753675942 | −0.04237875325990 |

Table S14: xyz coordinates of the acetonitrile−water structure in Å optimized at B3LYP-D3(BJ,abc)/def2-TZVP level using Orca 5.0.4.

| atom | x | y | z |
|---|---|---|---|
| C | −1.13435110533969 | −0.01321691422831 | 0.00143755723944 |
| N | 0.00921667916108 | −0.11366504306909 | 0.00171158867796 |
| C | −2.58193775332235 | 0.11621054014540 | 0.00102733451716 |
| H | −2.96073444065902 | 0.07785566950237 | 1.02273344798661 |
| H | −3.02988522341202 | −0.69537666589358 | −0.57300350672278 |
| H | −2.87068383046195 | 1.06726180285253 | −0.44732156456575 |
| H | 2.06187013782250 | 0.09784518722081 | −0.00666092824762 |
| O | 3.03050754889597 | 0.11435388769781 | −0.00746093408164 |
| H | 3.29066798731549 | −0.81142846422795 | 0.01678700519662 |

Table S15: xyz coordinates of the acrylonitrile−water structure in Å optimized at B3LYP-D3(BJ,abc)/def2-TZVP level using Orca 5.0.4.

| atom | x | y | z |
|---|---|---|---|
| C | 2.70617661634399 | 0.73455889475713 | −0.00895008435273 |
| C | 1.98505496518343 | −0.38438583760707 | 0.01588965888510 |
| H | 3.78665968902782 | 0.69338672625497 | 0.00468457797601 |
| H | 2.24016611834378 | 1.71027423915719 | −0.04312928284000 |
| H | 2.45295272164873 | −1.36110006726890 | 0.05042673690114 |
| C | 0.55986876164689 | −0.37929729058728 | −0.00008276980318 |
| N | −0.59110250395593 | −0.39344389087650 | −0.01232429848488 |
| H | −3.97427773901007 | −0.49428621941729 | −0.10377886300710 |
| O | −3.53617456592737 | 0.34880490629741 | 0.04712121786997 |
| H | −2.59031406330127 | 0.14110853929034 | 0.03313310685567 |

Table S16: xyz coordinates of the pyridine−water structure in Å optimized at B3LYP-D3(BJ,abc)/def2-TZVP level using ORCA 5.0.4.

| atom | x | y | z |
|---|---|---|---|
| C | −0.37318187575681 | −0.00999308135654 | −0.05385536915204 |
| C | 0.32717296728966 | −1.20866276231973 | −0.05080676891728 |
| C | 1.71320317606999 | −1.16737054380816 | 0.03387004153194 |
| N | 2.40195128129291 | −0.02777202596895 | 0.11138832078425 |
| C | 1.72347989743375 | 1.12022690285568 | 0.10970674753910 |
| C | 0.33829179592640 | 1.18000305233520 | 0.02839315119339 |
| H | −1.45393141045743 | −0.00230475650629 | −0.11785847189209 |
| H | −0.18560712219978 | −2.15932962141535 | −0.11116739892552 |
| H | 2.30057690409878 | −2.07880339392944 | 0.04305773916812 |
| H | 2.31472994927163 | 2.02669871967582 | 0.17760941776963 |
| H | −0.16671724311458 | 2.13672223813703 | 0.03101097588752 |
| H | 4.27737434038070 | −0.39401484941123 | 0.30074687739489 |
| O | 5.17970410706006 | −0.76586139208906 | 0.36357339659994 |
| H | 5.54328259670464 | −0.69249901819897 | −0.52400976698186 |

# References


1 Lüttschwager, N. NoisySignalIntegration.jl: A Julia Package for Uncertainty Evaluation of Numeric Integrals. *JOSS* **2021**, *6*, 3526.

2 Fischer, T. L.; Bödecker, M.; Schweer, S. M.; Dupont, J.; Lepère, V.; Zehnacker-Rentien, A.; Suhm, M. A.; Schröder, B.; Henkes, T.; Andrada, D. M. et al. The First HyDRA Challenge for Computational Vibrational Spectroscopy. *Phys. Chem. Chem. Phys.* **2023**, *25*, 22089–22102.

3 Fischer, T. L.; Wagner, T.; Gottschalk, H. C.; Nejad, A.; Suhm, M. A. A Rather Universal Vibrational Resonance in 1:1 Hydrates of Carbonyl Compounds. *J. Phys. Chem. Lett.* **2021**, *12*, 138–144.

4 Nedić, M.; Wassermann, T. N.; Larsen, R. W.; Suhm, M. A. A Combined Raman- and Infrared Jet Study of Mixed Methanol-Water and Ethanol-Water Clusters. *Phys. Chem. Chem. Phys.* **2011**, *13*, 14050–14063.

5 Cirtog, M.; Asselin, P.; Soulard, P.; Tremblay, B.; Madebène, B.; Alikhani, M. E.; Georges, R.; Moudens, A.; Goubet, M.; Huet, T. R.; Pirali, O.; Roy, P. The $(CH_2)_2O$-$H_2O$ Hydrogen Bonded Complex. Ab Initio calculations and Fourier Transform Infrared Spectroscopy from Neon Matrix and a New Supersonic Jet Experiment Coupled to the Infrared AILES Beamline of Synchrotron SOLEIL. *J. Phys. Chem. A* **2011**, *115*, 2523–2532.

6 Fischer, T. L. Of Resonances and Radicals – Hydrate Studies for Benchmarking, https://doi.org/10.53846/goediss-10364, PhD thesis. **2024**, accessed 04/15/2024.

7 Lwin, E.; Fischer, T. L.; Suhm, M. A. Microhydration of Tertiary Amines: Robust Resonances in Red-Shifted Water. *J. Phys. Chem. Lett.* **2023**, *14*, 10194–10199.

8 Gottschalk, H. C.; Poblotzki, A.; Fatima, M.; Obenchain, D. A.; Pérez, C.; Antony, J.; Auer, A. A.; Baptista, L.; Benoit, D. M.; Bistoni, G. et al. The First Microsolvation Step for Furans: New Experiments and Benchmarking Strategies. *J. Chem. Phys.* **2020**, *152*, 164303.

9 Mihrin, D.; Andersen, J.; Jakobsen, P. W.; Wugt Larsen, R. Highly Localized $H_2O$ Librational Motion as a Far-Infrared Spectroscopic Probe for Microsolvation of Organic Molecules. *Phys. Chem. Chem. Phys.* **2019**, *21*, 1717–1723.

10 Burevschi, E.; Peña, I.; Sanz, M. E. Geminal Diol Formation from the Interaction of a Ketone with Water in the Gas Phase: Structure and Reactivity of Cyclooctanone-$(H_2O)_{1,2}$ Clusters. *J. Phys. Chem. Lett.* **2021**, *12*, 12419–12425.

11 Emmeluth, C.; Dyczmons, V.; Kinzel, T.; Botschwina, P.; Suhm, M. A.; Yáñez, M. Combined Jet Relaxation and Quantum Chemical Study of the Pairing Preferences of Ethanol. *Phys. Chem. Chem. Phys.* **2005**, *7*, 991–997.

12 Andersen, J.; Heimdal, J.; Wugt Larsen, R. Spectroscopic Identification of Ethanol-Water

Conformers by Large-Amplitude Hydrogen Bond Librational Modes. *J. Chem. Phys.* **2015**, *143*, 224315.

13 Pugh, L. A.; Narahari Rao, K. Spectrum of Water Vapor in the 1.9 and 2.7$\mu$m Regions. *J. Mol. Spectrosc.* **1973**, *47*, 403–408.

14 Forney, D.; Jacox, M. E.; Thompson, W. E. The Mid- and Near-Infrared Spectra of Water and Water Dimer Isolated in Solid Neon. *J. Mol. Spectrosc.* **1993**, *157*, 479–493.

15 Larsen, R. W.; Zielke, P.; Suhm, M. A. Hydrogen-Bonded OH Stretching Modes of Methanol Clusters: A Combined IR and Raman Isotopomer Study. *J. Chem. Phys.* **2007**, *126*, 194307.

16 Mielke, Z.; Coussan, S.; Mierzwicki, K.; Roubin, P.; Sałdyka, M. The Complexes between $CH_3OH$ and $CF_4$. Infrared Matrix Isolation and Theoretical Studies. *J. Phys. Chem. A* **2006**, *110*, 4712–4718.

17 Perchard, J. P.; Romain, F.; Bouteiller, Y. Determination of Vibrational Parameters of Methanol from Matrix-Isolation Infrared Spectroscopy and Ab Initio Calculations. Part 1 – Spectral Analysis in the Domain 11000−200cm$^{-1}$. *Chem. Phys.* **2008**, *343*, 35–46.

18 Tanabe, S.; Ebata, T.; Fujii, M.; Mikami, N. OH Stretching Vibrations of Phenol−$(H_2O)_n$ (n=1–3) Complexes Observed by IR-UV Double-Resonance Spectroscopy. *Chem. Phys. Lett.* **1993**, *215*, 347–352.

19 Plokhotnichenko, A. M.; Radchenko, E. D.; Blagoi, Y. P.; Karachevtsev, V. A. Dimers of Phenol in Argon and Neon Matrices. *Low Temp. Phys.* **2001**, *27*, 666–675.

20 Emmeluth, C.; Dyczmons, V.; Suhm, M. A. Tuning the Hydrogen Bond Donor/Acceptor Isomerism in Jet-Cooled Mixed Dimers of Aliphatic Alcohols. *J. Phys. Chem. A* **2006**, *110*, 2906–2915.

21 Huisken, F.; Kaloudis, M.; Kulcke, A. Infrared Spectroscopy of Small Size-Selected Water Clusters. *J. Chem. Phys.* **1996**, *104*, 17–25.

22 Bouteiller, Y.; Perchard, J. P. The Vibrational Spectrum of $(H_2O)_2$: Comparison between Anharmonic Ab Initio Calculations and Neon Matrix Infrared Data Between 9000 and 90 $cm^{-1}$. *Chem. Phys.* **2004**, *305*, 1–12.

23 Dargent, D.; Madebène, B.; Soulard, P.; Tremblay, B.; Zins, E. L.; Alikhani, M. E.; Asselin, P. Conformational Landscape of the 1/1 Diacetyl/Water Complex Investigated by Infrared Spectroscopy and Ab Initio Calculations. *J. Phys. Chem. A* **2017**, *121*, 88–97.

24 Provencal, R. A.; Paul, J. B.; Roth, K.; Chapo, C.; Casaes, R. N.; Saykally, R. J.; Tschumper, G. S.; Schaefer, H. F. Infrared Cavity Ringdown Spectroscopy of Methanol Clusters: Single Donor Hydrogen Bonding. *J. Chem. Phys.* **1999**, *110*, 4258–4267.

25 Kollipost, F.; Papendorf, K.; Lee, Y.-F.; Lee, Y.-P.; Suhm, M. A. Alcohol Dimers - How Much Diagonal OH Anharmonicity? *Phys. Chem. Chem. Phys.* **2014**, *16*, 15948–15956.

26 Ebata, T.; Watanabe, T.; Mikami, N. Evidence for the Cyclic Form of Phenol Trimer: Vibrational Spectroscopy of the OH Stretching Vibrations of Jet-Cooled Phenol Dimer and Trimer. *J. Phys. Chem.* **1995**, *99*, 5761–5764.

27 Doi, A.; Mikami, N. Dynamics of Hydrogen-Bonded OH Stretches as Revealed by Single-Mode Infrared-Ultraviolet Laser Double Resonance Spectroscopy on Supersonically Cooled Clusters of Phenol. *J. Chem. Phys.* **2008**, *129*, 154308.

28 Kwasniewski, D.; Butler, M.; Reisler, H. Vibrational Predissociation of the Phenol-Water Dimer: A View from the Water. *Phys. Chem. Chem. Phys.* **2019**, *21*, 13968–13976.

29 Soulard, P.; Tremblay, B. Vibrational Study of Acrylonitrile Dimer and Acrylonitrile-Water Hydrogen-Bonded Complexes in Solid Neon Supported by Ab Initio Calculations. *J. Mol. Struct.* **2022**, *1266*, 133503.

30 Asselin, P.; Madebène, B.; Soulard, P.; Georges, R.; Goubet, M.; Huet, T. R.; Pirali, O.; Zehnacker-Rentien, A. Competition Between Inter- and Intra-Molecular Hydrogen Bonding: An Infrared Spectroscopic Study of Jet-Cooled Amino-Ethanol and its Dimer. *J. Chem. Phys.* **2016**, *145*, 224313.

31 Engdahl, A.; Nelander, B. The Intramolecular Vibrations of the Ammonia Water Complex. A Matrix Isolation Study. *J. Chem. Phys.* **1989**, *91*, 6604–6612.

32 Esteves-López, N.; Coussan, S. UV Photochemistry of Pyridine-Water and Pyridine-Ammonia Complexes Trapped in Cryogenic Matrices. *J. Mol. Struct.* **2018**, *1172*, 65–73.

33 Soulard, P.; Tremblay, B. Vibrational Study of Methylamine Dimer and Hydrated Methylamine Complexes in Solid Neon Supported by Ab Initio Calculations. *J. Mol. Struct.* **2021**, *1236*, 130308.

34 Bondi, A. Van der Waals Volumes and Radii. *J. Phys. Chem.* **1964**, *68*, 441–451.

35 Neese, F. Software Update: The ORCA Program System—Version 5.0. *Wiley Interdiscip. Rev. Comput. Mol. Sci.* **2022**, *12*.

36 Grimme, S. Exploration of Chemical Compound, Conformer, and Reaction Space with Meta-Dynamics Simulations Based on Tight-Binding Quantum Chemical Calculations. *J. Chem. Theory Comput.* **2019**, *15*, 2847–2862.

37 Pracht, P.; Bohle, F.; Grimme, S. Automated Exploration of the Low-Energy Chemical Space with Fast Quantum Chemical Methods. *Phys. Chem. Chem. Phys.* **2020**, *22*, 7169–7192.

38 Bannwarth, C.; Ehlert, S.; Grimme, S. GFN2-xTB-An Accurate and Broadly Parametrized Self-Consistent Tight-Binding Quantum Chemical Method with Multipole Electrostatics and Density-Dependent Dispersion Contributions. *J. Chem. Theory Comput.* **2019**, *15*, 1652–1671.
